# O-GlcNAcylation of PGK1 coordinates glycolysis and TCA cycle to promote tumor growth

**DOI:** 10.1038/s41467-019-13601-8

**Published:** 2020-01-07

**Authors:** Hao Nie, Haixing Ju, Jiayi Fan, Xiaoliu Shi, Yaxian Cheng, Xiaohui Cang, Zhiguo Zheng, Xiaotao Duan, Wen Yi

**Affiliations:** 1 MOE Key Laboratory of Biosystems Homeostasis & Protection, College of Life Sciences; The First Affiliated Hospital, School of Medicine, Zhejiang University, 310058 Hangzhou, China; 2 Department of Colorectal Surgery, Zhejiang Cancer Hospital, 310022 Hangzhou, China; 3 Division of Medical Genetics and Genomics, The Children’s Hospital, School of Medicine, Zhejiang University, 310058 Hangzhou, China; 4 State Key Laboratory of Toxicology and Medical Countermeasures, Beijing Institute of Pharmacology and Toxicology, 100850 Beijing, China

**Keywords:** Glycobiology, Cancer metabolism

## Abstract

Many cancer cells display enhanced glycolysis and suppressed mitochondrial metabolism. This phenomenon, known as the Warburg effect, is critical for tumor development. However, how cancer cells coordinate glucose metabolism through glycolysis and the mitochondrial tricarboxylic acid (TCA) cycle is largely unknown. We demonstrate here that phosphoglycerate kinase 1 (PGK1), the first ATP-producing enzyme in glycolysis, is reversibly and dynamically modified with O-linked N-acetylglucosamine (O-GlcNAc) at threonine 255 (T255). O-GlcNAcylation activates PGK1 activity to enhance lactate production, and simultaneously induces PGK1 translocation into mitochondria. Inside mitochondria, PGK1 acts as a kinase to inhibit pyruvate dehydrogenase (PDH) complex to reduce oxidative phosphorylation. Blocking T255 O-GlcNAcylation of PGK1 decreases colon cancer cell proliferation, suppresses glycolysis, enhances the TCA cycle, and inhibits tumor growth in xenograft models. Furthermore, PGK1 O-GlcNAcylation levels are elevated in human colon cancers. This study highlights O-GlcNAcylation as an important signal for coordinating glycolysis and the TCA cycle to promote tumorigenesis.

## Abstract (web summary)

Post-translational modifications of phosphoglycerate kinase 1 (PGK1), a glycoytic enzyme, contribute to cancer progression. Here, the authors show that PGK1 is O-GlcNAcylated at T255, which induces its translocation into mitochondria to suppress the tricarboxylic acid cycle for colorectal cancer growth.

## Introduction

Cancer cells display a high rate of glucose consumption and generate ATP from glycolysis followed by lactic acid fermentation, despite the availability of oxygen^1^. It is generally accepted that by switching the energy source from mitochondrial oxidative phosphorylation to glycolysis, cancer cells gain sufficient biomass-building materials for cell growth and proliferation, and combat highly oxidative microenvironment for better survival^2,3^. This metabolic reprogramming, also known as the Warburg effect, has shown to contribute to tumorigenesis in a variety of cancers, and become a promising target for diagnosis and targeted therapy of cancer^4,5^. However, how cancer cells coordinate glucose metabolism through glycolysis and mitochondrial TCA cycle is largely unknown.

O-GlcNAc is a prevalent post-translational modification (PTM) on serine and threonine residues of proteins. The addition and removal of O-GlcNAc on protein substrates are mediated by two enzymes, O-GlcNAc transferase (OGT) and O-GlcNAc hydrolase (OGA), respectively^6,7^. Increasing evidence has demonstrated that O-GlcNAcylation serves as a key player in regulating many important biological processes, including transcription, translation, metabolic reprogramming, and immune regulation^6,8^. Notably, O-GlcNAcylation has a complex interplay with phosphorylation, constituting an important mechanism to fine tune intracellular signaling^9,10^. Since O-GlcNAcylation is critical for normal physiology, its aberrant expression is closely associated with a number of diseases, including neurodegenerative diseases, diabetes, cardiovascular diseases, and cancers^8,11–13^. Emerging research evidence indicates that O-GlcNAcylation is globally elevated in various cancers, but the mechanisms involved in the tumor pathology remain incompletely understood at the molecular level^14,15^.

PGK1, the first ATP-generating enzyme in glycolysis, catalyzes the conversion of 1,3-diphosphoglycerate (1,3-BPG) to 3-phosphoglycerate (3-PG) and produces one molecule of ATP. PGK1 expression is upregulated in many types of human cancer, but the detail mechanisms by which PGK1 contributes to cancer development are not well understood^16,17^. PGK1 activity is mediated by different post-translational modifications. For example, phosphorylation of PGK1 at T243 and S203 positively regulates the Warburg effect and promotes the development of glioblastoma^17,18^. Acetylation at K220 inhibits PGK1 activity but is reversed by the activation of the insulin and mTOR pathway^19^. In addition, acetylation at K323 activates PGK1 enzyme activity and contributes to liver cancer progression^16^.

In this report, we identified an unknown regulatory mechanism of PGK1 by O-GlcNAcylation in cells. We observe that PGK1 is dynamically O-GlcNAcylated at T255, and this site-specific glycosylation enhances PGK1 activity and simultaneously induces PGK1 translocation into mitochondria. Inside mitochondria, PGK1 acts as a kinase to inhibit pyruvate dehydrogenase (PDH) complex to reduce pyruvate utilization and suppress oxidative phosphorylation. Blocking T255 O-GlcNAcylation on PGK1 suppresses the Warburg effect, decreases colon cancer cell proliferation, and inhibits tumor growth in nude mice. Moreover, O-GlcNAcylation of PGK1 is significantly elevated in human colon cancer tissues compared with the adjacent matching tissues.

## Results

### PGK1 expression is important for colon cancer development

**Fig. 1:**
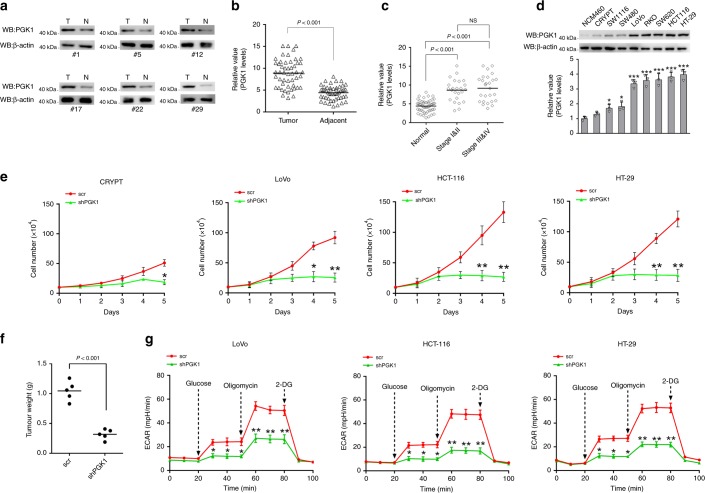
PGK1 expression is important for colon cancer development. **a** Immunoblotting analysis of PGK1 protein expression in 6 represented peritumoral/tumor tissue pairs from colon cancer patients. **b** Quantitative analysis of PGK1 expression showing significant increase in tumor tissues compared to the matching peritumoral tissues. **c** Quantitative analysis of PGK1 expression among different progression stages. **d** Immunoblotting analysis of PGK1 expression using a panel of colon cancer cell lines and two normal colon cell lines NCM460 and CRYPT. Quantitation of relative expression levels was shown. **e** Cell proliferation of CRYPT, LoVo, HCT-116 and HT-29 cells upon depletion of PGK1 using small hairpin RNA (shRNA). **f** Tumor weights measured at the experimental endpoint. HT-29 cells infected with control shRNA or shPGK1 were inoculated into the franks of nude mice (*n* = 5 per group). **g** The extracellular acidification rate (ECAR) in LoVo, HCT-116 and HT-29 cells infected with control shRNA or shPGK1. Each data point was the average of at least three independent measurements. Error bars denote the means ± standard deviations (SD). Statistical analyses were performed by one-way analysis of variance (ANOVA) followed by Tukey’s post hoc test (*P < 0.05, **P < 0.01, ***P < 0.001, NS = no significant difference). Source data are provided as a Source Date file.

Colon cancer is one of the leading causes of cancer mortality each year in both developed and developing countries. Colon cancer cells exhibit the typical Warburg effect. To investigate the possible role of PGK1 in colon cancer development and progression, we analyzed PGK1 protein expression levels in 50 peritumoral/tumor tissue pairs from colon cancer patients (Fig. 1a and Supplementary Fig. 1). Six pairs showed minimal PGK1 expression in either tumor or peritumoral tissues that prevented reliable quantitation. Among the remaining 44 pairs, 36 pairs showed relatively higher level of PGK1 in tumor tissues compared to the matching peritumoral tissues (Fig. 1a and Supplementary Fig. 1). Quantitation of the signals confirmed that the increase in PGK1 expression is statistically significant (*P* < 0.001 by Tukey’s post hoc test) (Fig. 1b). In addition, we classified the samples based on the progression of the disease, and compared the PGK1 expression levels. The PGK1 expression levels were increased significantly in all stages compared with the peritumoral tissues (Fig. 1c). However, there was no significant difference among different stages, suggesting that PGK1 may contribute to the development but not the progression of colon cancers.

Next, we analyzed PGK1 expression levels using a panel of colon cancer cell lines and two normal colon cell lines NCM460 and CRYPT (Fig. 1d). All of the seven colon cancer cell lines displayed higher PGK1 expression levels compared to the normal cell lines, which was consistent with high PGK1 expression in clinical tumor tissue samples. To investigate the possible role of PGK1 in colon cancer cells, we depleted PGK1 with small hairpin RNA (shRNA) in LoVo, HCT-116, and HT-29 cells, and observed a significant reduction in cell proliferation in all three cell lines (Fig. 1e and Supplementary Fig. 2). Depletion of PGK1 in the normal colon cell CRYPT also inhibited cell proliferation, but to a less extent compared to the cancer cell lines (Fig. 1e). To analyze the effect of PGK1 depletion on tumor growth, control (scramble shRNA) or PGK1-depleted HT-29 cells were injected into nude mice to determine the tumor formation ability. Consistently, PGK1-depleted HT-29 cells showed significantly reduced ability to form tumors as compared to control cells (Fig. 1f). These data suggest that high PGK1 expression is critical for colon cancer cell proliferation and tumor development. Since PGK1 is a key metabolic enzyme in glycolysis, we determined whether depletion of PGK1 impacted glucose metabolism in cells. As expected, PGK1 depletion caused a significant reduction in the extracellular acidification rate (ECAR) in LoVo, HCT-116 and HT-29 cells, suggesting a decreased glycolysis activity (Fig. 1g). However, PGK1 depletion had no significant effect on the oxygen consumption rate (OCR) in cells (Supplementary Fig. 3). A possible explanation is that upon the reduction of glycolysis, glutamine utilization is sustained or enhanced to fully support the TCA cycle metabolism as shown in previous reports^20–22^.

### PGK1 is O-GlcNAcylated at T255

**Fig. 2:**
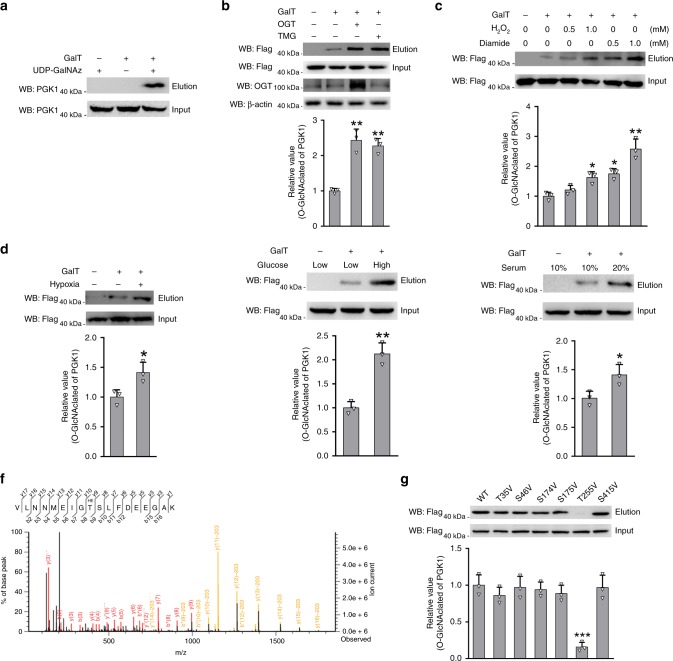
PGK1 is O-GlcNAcylated at T255. **a** Analysis of PGK1 O-GlcNAcylation in cells using a chemoenzymatic labeling method. O-GlcNAcylated proteins in HEK293T cell lysates were firstly labeled with an azido-N-acetylgalactosamine (GalNAz) sugar. Labeled proteins were then conjugated with biotin via Cu(I)-mediated [3 + 2] azide-alkyne cycloaddition (CuACC) chemistry and further captured with streptavidin-agarose beads. After stringent washing, the eluate was immunoblotted using an antibody against PGK1. **b** Analysis of PGK1 O-GlcNAcylation upon OGT overexpression or treatment with TMG. **c** Analysis of PGK1 O-GlcNAcylation upon cellular treatment with H_2_O_2_ or diamide. **d** Analysis of PGK1 O-GlcNAcylation in cell culture under hypoxia. **e** Analysis of PGK1 O-GlcNAcylation in cell culture under different glucose or serum concentrations. **f** Mapping the site of O-GlcNAcylation on PGK1 using mass spectrometry. **g** Probing the major site of glycosylation on PGK1 using various site-directed mutants. Error bars denote the means ± standard deviations (SD). Statistical analyses were performed by one-way analysis of variance (ANOVA) followed by Tukey’s post hoc test (*P < 0.05, **P < 0.01, ***P < 0.001). Source data are provided as a Source Date file.

Increasing evidence has shown that PTMs, such as phosphorylation, acetylation, and glycosylation, play an important role in regulating the structure and function of major glycolytic enzymes^23–25^. To investigate whether PGK1 is regulated by O-GlcNAcylation, we performed a well-established chemoenzymatic labeling experiment^25^. O-GlcNAcylated proteins in HEK293T cell lysates were firstly labeled with an azido-N-acetylgalactosamine (GalNAz) sugar. Labeled proteins were then conjugated with biotin via Cu(I)-mediated [3 + 2] azide-alkyne cycloaddition (CuACC) chemistry and further captured with streptavidin-agarose beads. After stringent washing, the eluate was immunoblotted using an antibody against PGK1. Immunoblotting signal was readily detected, but absent in control groups, indicating that PGK1 was O-GlcNAcylated in cells (Fig. 2a). As expected, PGK1 glycosylation was increased by about two-fold upon ectopic expression of OGT or treatment with thiamet-G (TMG), a specific inhibitor of OGA (Fig. 2b).

Cellular O-GlcNAcylation is highly dynamic in response to various environmental stimuli^26^. Thus, we investigated whether PGK1 glycosylation was also dynamically regulated when subjected to different metabolic stresses. Elevation of cellular levels of reactive oxygen species (ROS) by H_2_O_2_ or diamide (a thiol-oxidizing compound) increased PGK1 glycosylation in a dose-dependent manner (Fig. 2c and Supplementary Fig. 4). Hypoxia, a common form of metabolic stress in solid tumors, has been shown to induce ROS generation at ETC complex III in mitochondria^27,28^. Thus, hypoxia was shown to induce PGK1 glycosylation about 3-fold compared to the normoxic condition (Fig. 2d). In addition, PGK1 glycosylation was elevated in the high glucose medium or with high serum concentrations (Fig. 2e). Together these data show that O-GlcNAcylation of PGK1 is dynamically regulated by different cellular metabolic stresses, suggesting a signaling role in cells.

To identify the site(s) of O-GlcNAcylation on PGK1, we transiently co-expressed Flag-tagged PGK1 and OGT in HEK293T cells. After immunoprecipitation using anti-Flag M2 beads and in-gel trypsin digestion, resulted peptides were subjected to mass spectrometry analysis^29^. We identified six putative O-GlcNAcylation sites (T35, S46, S174, S175, T255, S415) on PGK1 (Fig. 2f, and Supplementary Fig. 5). To determine which residue(s) is the major glycosylation site in PGK1, we mutated each of the six residues to valine. We found that mutation of T255, but not the other five residues, reduced the O-GlcNAcylation signal to a large degree, suggesting that T255 is the major glycosylation site (Fig. 2g).

### T255 O-GlcNAcylation activates PGK1

**Fig. 3:**
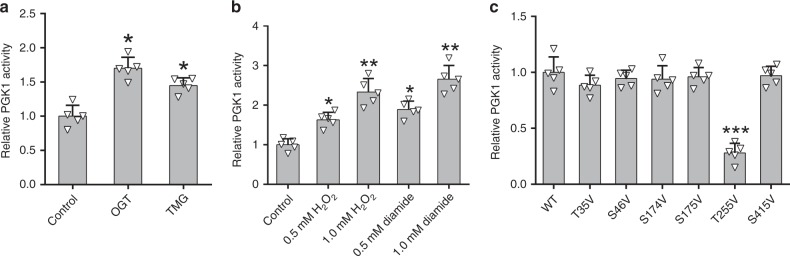
T255 O-GlcNAcylation activates PGK1 enzyme activity. **a** Enzymatic activity of PGK1 under basal conditions, upon OGT overexpression or with TMG treatment. **b** Enzymatic activity of PGK1 upon cellular treatment with different concentrations of H_2_O_2_ or diamide. **c** Comparison of enzymatic activity of PGK1 WT and different mutants upon OGT overexpression. Error bars denote the means ± standard deviations (SD). Statistical analyses were performed by one-way analysis of variance (ANOVA) followed by Tukey’s post hoc test (*P < 0.05, **P < 0.01, ***P < 0.001). Source data are provided as a Source Date file.

PGK1 catalyzes the first ATP-yielding step in glycolysis, and is important for coordinating energy production with biosynthesis and redox balance. Thus, we investigated the role of T255 glycosylation in PGK1. In vitro enzymatic assays showed that enhancing O-GlcNAcylation in cells by OGT overexpression or TMG treatment induced PGK1 activity by 1.8 and 1.5-fold, respectively (Fig. 3a). H_2_O_2_ or diamide treatment enhanced PGK1 activity in a dose-dependent manner, positively correlated with the induction of glycosylation levels (Fig. 3b). To further confirm the inducing effect of glycosylation, we analyzed the activity of various PGK1 mutants. Consistent with the site mapping results, we observed that upon OGT overexpression, T255V mutant, but not other mutants, displayed a significant decrease in PGK1 activity by about 72% compared to the WT (Fig. 3c). Thus, T255 glycosylation activates PGK1 activity.

To further understand the effect of O-GlcNAcylation on PGK1 activity, we determined the steady-state kinetics of PGK1-catalyzed reactions (Supplementary Tables 1 and 2). PGK1 T255V had a higher *K*_m_ value (7.50 µM) for 3-PG than PGK1 WT (4.77 µM). OGT overexpression had no significant effect on *K*_m_ values for 3-PG with either T255V or WT protein. Conversely, PGK1 T255V had a two-fold lower catalytic efficiency (*K*_cat_/*K*_m_ = 2.3) for 3-PG than PGK1 WT (*K*_cat_/*K*_m_ = 4.94). OGT overexpression further increased the catalytic efficiency (*K*_cat_/*K*_m_ = 12.55) by 2.5-fold compared to PGK1 WT, but had no apparent effect on the *K*_cat_/*K*_m_ value with T255V mutant. Since PGK1 catalyzes a reversible reaction, we then determined the kinetic parameters for the forward reaction. PGK1 T255V had a higher *K*_m_ value (13.92 µM) for ADP than PGK1 WT (7.69 µM). OGT overexpression decreased *K*_m_ value for ADP at about 2-fold with PGK1 WT, but had no obvious effect with PGK1 T255V. Conversely, PGK1 T255V had a 2.4-fold lower catalytic efficiency (*K*_cat_/*K*_m_ = 8.6) for ADP than PGK1 WT (*K*_cat_/*K*_m_ = 20.28). OGT overexpression further increased the catalytic efficiency (*K*_cat_/*K*_m_ = 12.71) by 4.4-fold as with PGK1 WT, but only had a slight effect on the *K*_cat_/*K*_m_ value as with PGK1 T255V. Thus, O-GlcNAcylation appears to activate PGK1-catalyzed reactions from both directions, and enhances the substrate binding affinity.

Since proteins obtained from 293T cells could contain multiple PTMs, we want to confirm that differences in steady-state kinetics does not stem from intrinsic changes in the protein stability or structure. We expressed and purified His-tagged WT and T255V PGK1 from E. coli, and determined the enzymatic activity and the kinetic parameters (Supplementary Fig. 6). The results showed that there was a slight decrease in the activity of PGK1 T255V compared to the WT, but the difference was not significant. The steady-state kinetics parameters were similar between WT and T255V PGK1(Supplementary Tables 3 and 4). Thus, these data indicate that T255V mutation does not cause apparent perturbations to the protein stability and function.

### O-GlcNAcylation of PGK1 is important for colon cancer cell proliferation

**Fig. 4:**
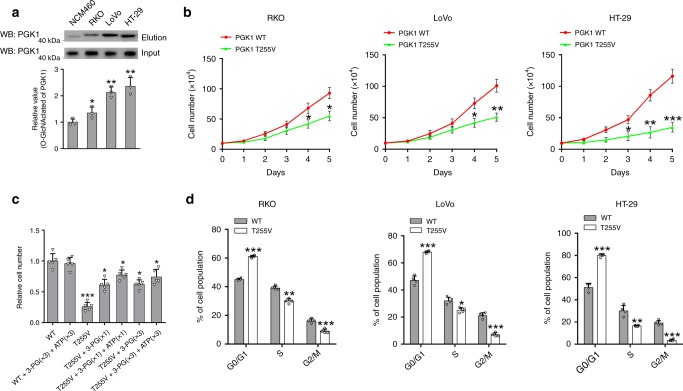
O-GlcNAcylation of PGK1 is important for colon cancer cell proliferation. **a** Analysis of PGK1 glycosylation in a panel of three human colon cancer cell lines and a normal colon cell line NCM460. **b** Cell proliferation of PGK1 WT or T255V rescue cells. In RKO, LoVo and HT-29 cells, endogenous PGK1 was depleted with shRNA targeting PGK1, and then rescued with ectopic expression of PGK1 WT or PGK1 T255V, which is resistant to shRNA targeting. **c** Cell proliferation of PGK1 WT or T255V rescue HT-29 cells in the presence of various 3-PG and ATP concentrations. **d** Cell cycle analysis of PGK1 WT or T255V rescue RKO, LoVo and HT-29 cells. Error bars denote the means ± standard deviations (SD). Statistical analyses were performed by one-way analysis of variance (ANOVA) followed by Tukey’s post hoc test (*P < 0.05, **P < 0.01, ***P < 0.001). Source data are provided as a Source Date file.

To gain a better understanding of the impact of PGK1 O-GlcNAcylation on colon cancer, we first examined the glycosylation levels using a panel of three human colon cancer cell lines and a normal colon cell line NCM460. PGK1 glycosylation was observed elevated in all three colon cancer cell lines compared to NCM460 (Fig. 4a). In addition, PGK1 glycosylation was observed across different human cancer cell lines, although with various levels (Supplementary Fig. 7).

To investigate the importance of T255 O-GlcNAcylation, we knocked down endogenous PGK1 expression and then rescued PGK1 expression with PGK1 WT or T255V, which is resistant to shRNA targeting. PGK1 WT and T255V were expressed at a comparable level in cells (Supplementary Fig. 8a). Furthermore, PGK1 WT and T255V displayed a similar half-life in cells, indicating that T255V mutant did not affect protein stability (Supplementary Fig. 8b). Cell proliferation rates were then determined for each rescue cell line. We found that PGK1 T255V rescue RKO, LoVo and HT-29 cells showed a significant decrease in cell proliferation compared to their corresponding PGK1 WT rescue cells (Fig. 4b), suggesting that T255 glycosylation is functionally important in colon cancer cells.

To understand why T255V mutation inhibits cell proliferation, we first supplemented 3-PG and ATP^30,31^, the primary products of PGK1-catalyzed reaction in the culture medium, which resulted in a partial rescue of the cell proliferation (Fig. 4c). Increasing 3-PG or ATP concentrations still did not fully rescue the cell proliferation, indicating that decreasing PGK1 enzyme activity by T255V mutation did not solely account for the inhibition. PGK1 T255V rescue HT-29 cells showed enhanced levels of ROS compared to the WT rescue cells. Addition of *N*-acetyl cysteine (NAC) to the culture medium reduced ROS levels in T255V rescue cells, but failed to rescue the proliferation (Supplementary Fig. 9). Thus, elevation in ROS levels did not account for the inhibition of cell proliferation. We then determined whether T255V affects cell cycle regulation. Compared to WT rescue cells, T255V rescue cells showed enhanced percentage of G0/G1 phase but decreased percentage of S and G2/M phases (Fig. 4d), indicating that T255V mutation caused a cell-cycle arrest at G0/G1 phase.

### T255 O-GlcNAcylation mediates mitochondrial translocation of PGK1

**Fig. 5:**
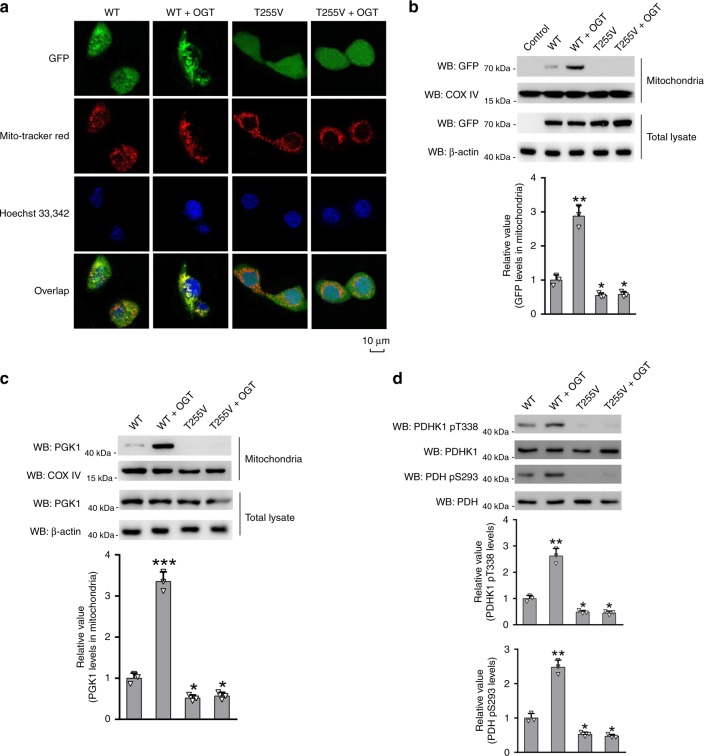
T255 O-GlcNAcylation induces the mitochondrial translocation of PGK1. **a** Cellular distribution of PGK1 as determined by immunostaining in the presence or absence of OGT overexpression. HT-29 cells were transfected with GFP-fused PGK1 WT or T255V construct. Cells were co-stained with MitoTracker red, a fluorescent marker for mitochondria. **b**, **c** Immunoblotting analysis of cellular distribution of PGK1 WT or T255V in the presence or absence of OGT overexpression. **d** Immunoblotting analysis of PDHK1 and PDH phosphorylation in mitochondria. Mitochondrial fractions were prepared and immunoblotted with indicated antibodies. Total cell lysates were used as a control. Error bars denote the means ± standard deviations (SD). Statistical analyses were performed by one-way analysis of variance (ANOVA) followed by Tukey’s post hoc test (*P < 0.05, **P < 0.01, ***P < 0.001). Source data are provided as a Source Date file.

Since PGK1 enzymatic activity did not solely account for the inhibition of cell proliferation by T255V mutation, we investigated whether there exist other mechanisms. In a previous study, PGK1 was reported to translocate into mitochondria mediated by S203 phosphorylation^17^. Once inside mitochondria, PGK1 acted as a protein kinase to phosphorylate and activate pyruvate dehydrogenase kinase 1 (PDHK1), which subsequently phosphorylates and deactivates pyruvate dehydrogenase (PDH). This results in the suppression of mitochondrial pyruvate metabolism, and enhancement of the Warburg effect and cell proliferation^17^. Therefore, we investigated whether T255 O-GlcNAcylation also mediated PGK1 translocation into mitochondria. We transfected HT-29 cells with GFP-fused PGK1 WT or PGK1 T255V construct to track the cellular distribution of PGK1. Cells were co-stained with MitoTracker red, a fluorescent marker for mitochondria (Fig. 5a). The results showed that GFP-PGK1 WT partially co-localized with mitochondria. OGT overexpression enhanced the co-localization signal. In contrast, GFP-PGK1 T255V did not show any detectable co-localization with mitochondria, and OGT overexpression had no apparent effect. Cell fractionation and immunoblotting analysis also confirmed that in PGK1 WT expressing cells, GFP signal was greatly elevated in the mitochondrial fraction in the presence of OGT overexpression. Whereas in PGK1 T255V expressing cells, GFP signal was not detected (Fig. 5b). As another confirmation, HT-29 stable cells expressing PGK1 WT or PGK1 T255V rescue construct were analyzed. Immunoblotting with the anti-PGK1 antibody showed similar results (Fig. 5c). Next, the downstream effect of PGK1 mitochondrial translocation was analyzed. Consistent with the previous study, we showed that mitochondrial translocation of PGK1 by OGT overexpression significantly increased T338 phosphorylation of PDHK1, and S293 phosphorylation of PDH in PGK1 WT rescue cells, but not in PGK1 T255V rescue cells (Fig. 5d). Thus, these data indicate that T255 O-GlcNAcylation promotes the mitochondrial translocation of PGK1 in cells.

**Fig. 6:**
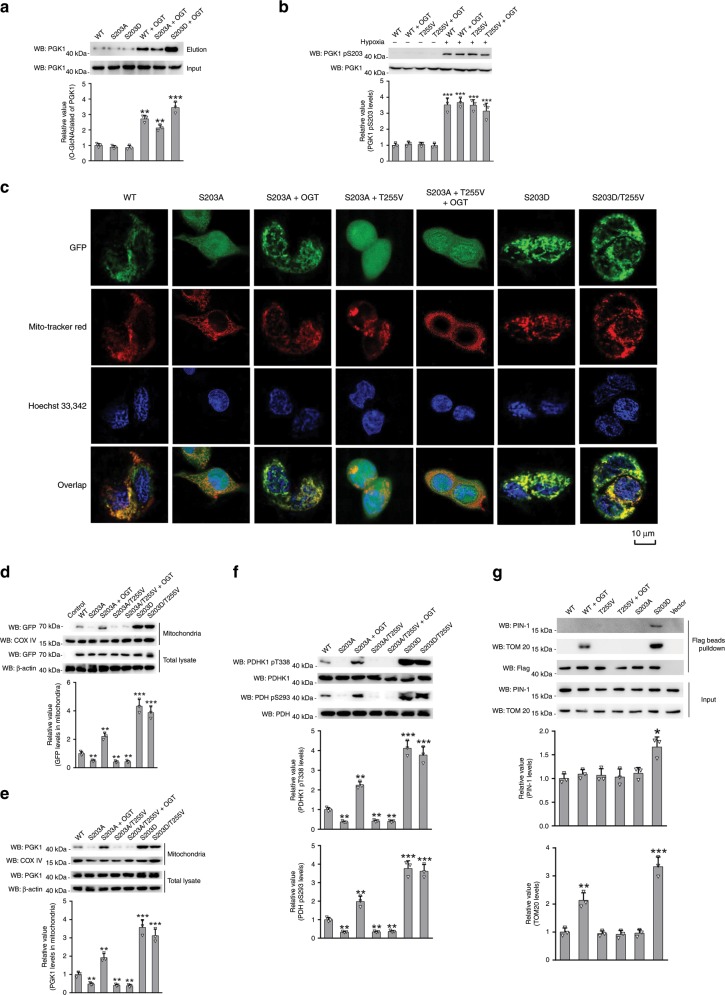
T255 glycosylation mediates the mitochondrial translocation of PGK1 independent of S203 phosphorylation. **a** Immunoblotting analysis of PGK1 O-GlcNAcylation in HT-29 cells stably expressing PGK1 S203D or S203A mutants in the presence or absence of OGT overexpression. **b** Immunoblotting analysis of PGK1 S203 phosphorylation in HT-29 cells rescued with PGK1 WT or T255V mutant, in the presence or absence of hypoxia. **c** Cellular distribution of different PGK1 mutants as determined by immunostaining. **d**, **e** Immunoblotting analysis of cellular distribution of different PGK1 mutants. Mitochondrial fractions were prepared and immunoblotted with indicated antibodies. **f** Immunoblotting analysis of PDHK1 and PDH phosphorylation in mitochondria. Mitochondrial fractions were prepared and immunoblotted with indicated antibodies. **g** Interaction of PGK1 WT or mutants with PIN-1 or TOM20 as shown by co-immunoprecipitation. Error bars denote the means ± standard deviations (SD). Statistical analyses were performed by one-way analysis of variance (ANOVA) followed by Tukey’s post hoc test (*P < 0.05, **P < 0.01, ***P < 0.001). Source data are provided as a Source Date file.

O-GlcNAcylation is known to have a complex crosstalk with protein phosphorylation^9,32^. The observation that both T255 O-GlcNAcylation and S203 phosphorylation contribute to mitochondrial translocation of PGK1 prompted us to investigate the interplay between these two forms of modifications. We generated PGK1 S203A (phosphorylation deficient mutant) and S203D (phosphorylation mimetic mutant) rescue HT-29 cells to probe the effects on T255 O-GlcNAcylation. As shown in Fig. 6a, both PGK1 S203A and PGK1 S203D displayed comparable glycosylation levels compared to PGK1 WT, suggesting that mutations on S203 did not affect the basal level of T255 O-GlcNAcylation. However, in the presence of OGT overexpression, PGK1 S203D displayed a higher glycosylation level than either PGK1 WT or PGK1 S203A, suggesting that S203 phosphorylation might render PGK1 to become a better substrate for OGT-catalyzed reactions. On the other hand, when we probed S203 phosphorylation in PGK1 WT or PGK1 T255V rescue cells, no detectable signals were observed under normal cultured conditions (with 20% O_2_), with or without OGT overexpression (Fig. 6b). Under hypoxic conditions which were shown to induce S203 phosphorylation, PGK1 WT and PGK1 T255V displayed comparable S203 phosphorylation levels (Fig. 6b). OGT overexpression had no apparent effect on S203 phosphorylation under hypoxic conditions. Therefore, T255 O-GlcNAcylation and S203 phosphorylation are independent modification events.

Next we investigate whether T255 glycosylation and S203 phosphorylation independently mediate PGK1 translocation. Consistent with the previous study, PGK1 S203A alone did not show detectable co-localization with mitochondria, and PGK1 S203D showed clear co-localization signals (Fig. 6c). However, OGT overexpression significantly induced the co-localization signal of PGK1 S203A within mitochondria, suggesting that glycosylation could induce PGK1 translocation in the absence of S203 phosphorylation. On the other hand, PGK1 S203D/T255V showed a comparable co-localization signal as PGK1 S203D, suggesting that S203 phosphorylation could induce PGK1 translocation in the absence of T255 glycosylation. Consistently, elimination of both S203 phosphorylation and T255 glycosylation (S203A/T255V construct) abolished the co-localization signal with or without OGT overexpression (Fig. 6c). We also performed experiments under hypoxic conditions (Supplementary Fig. 10a). Hypoxia was shown to induce both S203 phosphorylation and T255 glycosylation. Elimination of single modification (S203A or T255V) did not abolish the co-localization signal, while the double mutation construct did (Supplementary Fig. 10a). The subsequent cell fractionation and immunoblotting analysis further confirmed the immunostaining results (Fig. 6d, e, and Supplementary Fig. 10b–c). Moreover, the downstream effect of PGK1 mitochondrial translocation (i.e, PDHK1 T338 phosphorylation and PDH S293 phosphorylation) was also consistent with the translocation of PGK1 variants (Fig. 6f, and Supplementary Fig. 10d). Taken together, these data indicate that S203 phosphorylation and T255 O-GlcNAcylation independently mediate the mitochondrial translocation of PGK1.

Finally we probed the mechanism by which T255 glycosylation induced PGK1 translocation. The previous study showed that PGK1 S203 phosphorylation was recognized by the peptidyl-proline isomerase protein never in mitosis gene A interacting-1 (PIN-1), which facilitated the interaction of PGK1 with the translocase of the outer membrane complex (TOM20) for the subsequent mitochondrial translocation^17^. Using co-immunoprecipitation (CoIP) assays, we showed that neither PGK1 WT nor T255V interacted with PIN-1 in the presence or absence of OGT overexpression, suggesting that T255 glycosylation is not required for PGK1/PIN-1 interaction induced by S203 phosphorylation (Fig. 6g). However, under OGT overexpression PGK1 WT, but not T255V, interacted with TOM20, similar to S203D which served as a positive control in the assay (Fig. 6g). Thus, O-GlcNAcylation appears to increase the interaction of PGK1 with TOM20 to facilitate the translocation of PGK1 into mitochondria.

### T255 O-GlcNAcylation of PGK1 regulates glucose metabolism in cells

**Fig. 7:**
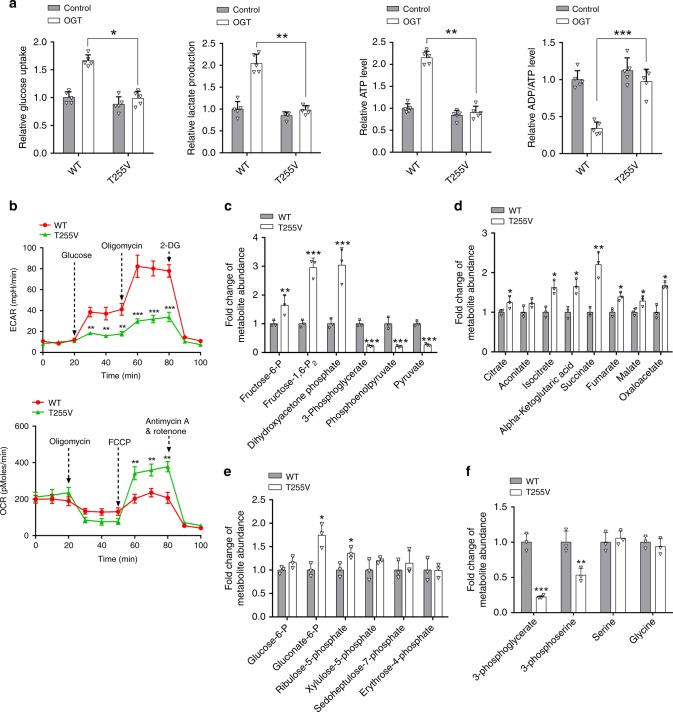
T255 O-GlcNAcylation of PGK1 regulates glucose metabolism in cells. **a** Comparison of glucose uptake, lactate production, ATP levels, and the total energy charge (ADP/ATP ratio) in PGK1 WT or T255V rescue HT-29 cells in the presence or absence of OGT overexpression. **b** The extracellular acidification rate (ECAR) and oxygen consumption rate (OCR) in PGK1 WT or T255V rescue HT-29 cells in the presence of OGT overexpression. **c**–**f** Relative abundance of metabolites derived from glycolysis (**c**), TCA cycle (**d**), the pentose phosphate pathway (**e**), and the serine synthetic pathway (**f**) in PGK1 WT or T255V rescue HT-29 cells in the presence of OGT overexpression. Error bars denote the means ± standard deviations (SD). Statistical analyses were performed by one-way analysis of variance (ANOVA) followed by Tukey’s post hoc test (*P < 0.05, **P < 0.01, ***P < 0.001). Source data are provided as a Source Date file.

According to the Warburg effect, proliferating cancer cells preferentially use glycolysis over mitochondrial oxidative phosphorylation for glucose-dependent ATP production. They consume a large amount of glucose, maintain a high rate of glycolysis and convert the majority of glucose into lactic acid. Our results demonstrated that O-GlcNAcylation not only increased PGK1 enzymatic activity, but also induced PGK1 translocation into mitochondria to deactivate PDH, suggesting that PGK1 O-GlcNAcylation may play an important role in regulating glucose metabolism through glycolysis and the TCA cycle. As shown in Fig. 7a, while glucose uptake, lactate production and ADP/ATP ratio were comparable between PGK1 WT and T255V rescue HT-29 cells, upon OGT overexpression, glucose uptake and lactate production were significantly increased, and ADP/ATP ratio was significantly decreased, in PGK1 WT rescue cells compared to PGK1 T255V rescue cells. Consistently, in the presence of OGT overexpression, the ECAR was significantly upregulated, and the OCR was significantly downregulated, in PGK1 WT rescue cells compared to PGK1 T255V rescue cells (Fig. 7b).

To further corroborate these results, we performed relative quantification of cellular metabolites using liquid chromatography coupled with mass spectrometry (LC-MS) analysis. We found that in the presence of OGT overexpression, PGK1 T255V rescue cells accumulated higher levels of fructose-1,6-diphosphate and dihydroxyacetone phosphate, but lower levels of 3-PG, phosphoenolpyruvate, and pyruvate, compared to PGK1 WT rescue cells, suggesting a relatively decreased glycolytic activity (Fig. 7c). In contrast, the levels of a number of metabolites specific in the TCA cycle were higher in PGK1 T255V rescue cells (Fig. 7d). The levels of metabolites involved in the pentose phosphate pathway (PPP) were not significantly altered except gluconate-6-phosphate (Fig. 7e). In addition, the level of 3-phosphoserine, a key metabolite involved in the de novo serine synthesis pathway (SSP), was significantly decreased in PGK1 T255V rescue cells (Fig. 7f). This might result from the reduction of 3-PG concentration, the entering point of the SSP, thus suggesting a decrease of metabolism in the SSP. The cellular levels of serine and glycine were not changed, probably because cells could take up serine and glycine from the culture medium. Collectively, T255 O-GlcNAcylation regulates glucose metabolism through the major metabolic pathways.

### T255 O-GlcNAcylation of PGK1 promotes tumor formation and increases in colon tumor tissues

**Fig. 8:**
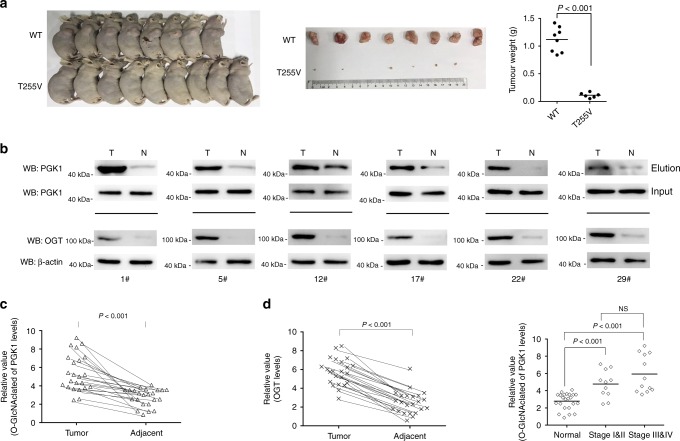
T255 O-GlcNAcylation of PGK1 promotes tumor formation and increases in colon tumor tissues. **a** Tumor formation in nude mice injected with PGK1 WT or T255V rescue HT-29 cells. Dissected tumors at the experimental endpoint were shown, and the masses of the dissected tumors were weighted. **b** Immunoblotting analysis of PGK1 glycosylation and OGT expression in human colon tumor (T) tissues and the matching adjacent tissues (N). **c**–**e** The statistical analysis of paired tumor and matching adjacent tissues. Relative PGK1 glycosylation level was normalized to the total PGK1 protein level for each patient. Source data are provided as a Source Date file.

To investigate whether PGK1 glycosylation contributes to tumorigenesis in vivo, we performed mouse xenograft studies. PGK1 WT or T255V rescue HT-29 cells were subcutaneously injected into immuno-compromised mice, and analyzed for their ability to form tumors. We observed that mice injected with PGK1 T255V rescue cells produced much smaller tumors compared to mice injected with PGK1 WT rescue cells (Fig. 8a). The results are keeping with the in vitro data and showed that glycosylation of PGK1 on T255 is critical for tumor growth in vivo.

To probe the clinical relevance of PGK1 glycosylation, we examined O-GlcNAcylation levels of PGK1 in human colon cancer tissues. A total of 50 pairs of primary human colon cancer tissue samples with the matched adjacent normal tissues were collected, and PGK1 glycosylation levels were determined. Six pairs of samples were excluded as they showed minimal PGK1 expression to prevent reliable quantification. We observed that in the remaining samples, 23 pairs exhibited relatively higher level of PGK1 glycosylation in cancer tissues (Fig. 8b and Supplementary Fig. 11). Quantification confirmed that the increase in the level of PGK1 glycosylation is statistically significant (Fig. 8c). We further examined OGT expression in these samples. We observed that 27 pairs of samples showed higher levels of OGT expression in cancer tissues compared to the matched tissues (Fig. 8b, d, and Supplementary Fig. 11), indicating that OGT expression positively correlates with PGK1 glycosylation levels in colon cancer. To determine whether PGK1 O-GlcNAcylation correlates with colon cancer progression, we grouped the 23 paired samples according to the stages and compared PGK1 glycosylation levels between different groups. The levels of PGK1 O-GlcNAcylation were significantly higher across all stages of the disease compared to the normal tissues. However, there was no significant difference among different stages (Fig. 8e). Thus, our data suggest that PGK1 O-GlcNAcylation may be critical for colon cancer development.

## Discussion

**Fig. 9:**
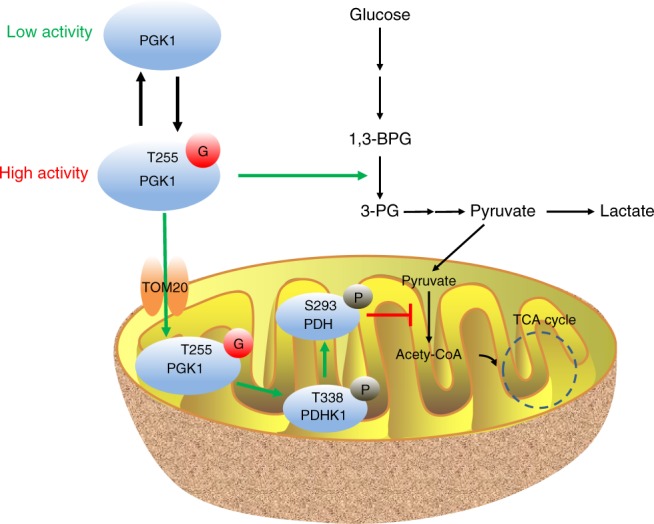
A graphic model of PGK1 O-GlcNAcylation in coordination of glycolysis and TCA cycle to promote tumor growth. T255 O-GlcNAcylation activates PGK1 enzymatic activity, and simultaneously induces PGK1 translocation into mitochondria for the subsequent phosphorylation-dependent inhibition of pyruvate metabolism. Thus, PGK1 O-GlcNAcylation promotes the Warburg effect and contributes to colon cancer tumorigenesis.

Proliferating cancer cells are known to exhibit aberrant glucose metabolism characterized by elevated glucose uptake and a high rate of glycolysis^33,34^. The increased metabolic flux through glycolysis and suppressed metabolism of the TCA cycle have been demonstrated to provide biosynthetic precursors for rapid macromolecule synthesis, and to maintain cellular redox homeostasis for better survival^35,36^. However, how cancer cells coordinate glucose metabolism between glycolysis and the TCA cycle is largely unclear. Here, we identified a previously unknown mechanism for regulation of glucose metabolism through major metabolic pathways. PGK1, the first ATP-generating enzyme in glycolysis, is dynamically regulated by O-GlcNAcylation. Glycosylation on T255 activates its enzymatic activity, and simultaneously induces PGK1 translocation into mitochondria for the subsequent phosphorylation-dependent inhibition of pyruvate metabolism (Fig. 9). Coordination of glycolysis and mitochondrial metabolism through the regulation of PGK1 glycosylation promotes cell proliferation and tumorigenesis.

PTMs play critical roles in regulating the structure and function of many metabolic enzymes, thus serving as an important mechanism to regulate cellular metabolism. O-GlcNAcylation has been demonstrated to participate in regulating many biological processes in cells. Its role in regulating cell metabolism has just begun to elucidate. Recently, two key regulatory enzymes in glycolysis, phosphofrutokinase 1 and pyruvate kinase, were shown to be regulated by O-GlcNAcylation^25,37^. O-GlcNAcylation of these two enzymes redirects glucose metabolism to anabolic pathways to meet the high biosynthetic demands of proliferating cancer cells. In our previous study, O-GlcNAcylation of glucose-6-phosphate dehydrogenase, the rate-limiting enzyme in the pentose phosphate pathway, enhanced cellular production of ribose-5-phosphate and NADPH, which promoted nucleotide biosynthesis and anti-oxidative response^29^. Collectively, studies from our lab and others convincingly demonstrate that O-GlcNAcylation emerges as an important mechanism to coordinate glucose flux through major metabolic pathways to promote cell proliferation and tumor growth in vivo. It is expected that further studies will uncover a broader role of O-GlcNAcylation in regulating cell metabolism.

PGK1 has been shown in previous studies to possess different PTMs including phosphorylation and acetylation^18,19,38^. These site-specific modifications are differentially regulated by various stimuli or the tumor microenvironment. Among the reported phosphorylation sites, S203 phosphorylation plays a key role in mediating the translocation of PGK1 into mitochondria for the subsequent inhibition of pyruvate metabolism and ROS production^17^. In our study, we demonstrate that T255 glycosylation and S203 phosphorylation are independent modification events on PGK1, and that T255 glycosylation serves as an independent signal to induce PGK1 translocation into mitochondria. During cell growth and proliferation, cells must adapt to a dynamically changing environment, which varies in nutrients, oxygen, and other signaling cues. It is likely that different PTMs have a unique response to certain stimuli, thus ensuring that cells can effectively coordinate metabolic regulation with the changing environment to maximize the ability to survive and thrive.

Altered metabolism has recently emerged as an important target for anti-cancer therapy in both preclinical and clinical studies^39–41^. The strategy to target key metabolic enzymes should be executed with caution, because they are also critical for the normal cell physiology. PTMs constitute a higher level of regulation of metabolic enzymes, and have been shown significantly altered in the tumor settings. Thus, targeting altered PTMs to normalize the metabolic flux might provide a more viable alternative strategy. Studies are ongoing in our lab to investigate the notion to target O-GlcNAcylated metabolic enzymes in tumor.

## Methods

### Cell culture

Cell lines HEK293T, GES-1, LO2, PANC-1, MGC-803, HepG2, HT-29, U251, MCF-7, H1299, ACHN, NCM460, CRYPT, LoVo, HCT-116 and RKO were all obtained from ATCC. Cell lines were routinely cultured in Roswell Park Memorial Institute (RPMI) 1640 medium (Hyclone) or high glucose Dulbecco’s Modified Eagle Medium (DMEM) medium (Hyclone), which contained 10% fetal calf serum (Gibco), 100 U/mL penicillin, and 100 U/mL streptomycin, in a humidified cell incubator at 37 °C with an atmosphere of 5% CO_2_.

### Human specimens

Small pieces (~0.5cm^3^) of colon tumor tissues and the matching tumor-adjacent tissues from the same patient were taken from surgically resected colon specimens as part of the colon cancer biobanking process at the Zhejiang Cancer Hospital Bio-specimen Repository (Hangzhou, China). All patients were registered at Zhejiang Cancer Hospital (Hangzhou, China) and the informed consent was obtained from each patient before the study. These specimens were examined and diagnosed by pathologists at Zhejiang Cancer Hospital. The research protocol was approved by the Ethic Committee of the School of Medicine, Zhejiang University (Hangzhou, China). The entire experimental protocol was conducted in compliance with the institutional guidelines.

### Western blotting analysis

Approximately 30 μg of lysed protein was separated by 8–15% sodium dodecyl sulfate PAGE, transferred to a nitrocellulose membrane (Bio-Rad), and then blocked in the blocking buffer (5% bovine serum albumin solution and 0.1% Tween 20 in Tri-buffered saline (TBST)). The membrane was incubated with the indicated antibodies. Antibodies used in this study were obtained from the following sources: anti-PGK1 antibody (1:1000, Santa Cruz, sc-166432), anti-Flag antibody (1:1000, Sigma-Aldrich, SAB4200071), anti-OGT antibody (1:1000, Cell Signaling Technology, #24083), anti-PDHK1 antibody (1:1000, Cell Signaling Technology, #3820), anti-PDHK1 (Phospho-Thr338) antibody (1:1000, Signalway Antibody, 11596), anti-TOM20 antibody (1:1000, Cell Signaling Technology, #42406), anti-PIN1 antibody (1:1000, Cell Signaling Technology, #3722), anti-PDH antibody (1:1000, Cell Signaling Technology, #3205), anti-PDH (Phospho-Ser293) antibody (1:1000, Biobyt, orb6670), anti-β-actin antibody (1:1000, Cell Signaling Technology, #4970), anti-COX IV antibody (1:1000, Cell Signaling Technology, #4850), anti-GFP antibody (1:1000, Cell Signaling Technology, #2956), anti-His antibody (1:1000, Cell Signaling Technology, #12698) and anti-PGK1(Phospho-Ser203) antibody (2.0 μg/mL, generated by Abgent). Proteins were visualized using an Odyssey Infrared Imaging System (LI-COR). Uncropped and unprocessed scans of the blots are supplied in the Source Data file.

### PGK1 O-GlcNAcylation analysis

The labeling and detection of PGK1 glycosylation were carried out as previously described^25^. Briefly, the cell lysate was labeled using a chemoenzymatic method, in which GalT (Y289L) was used to transfer UDP-GalNAz onto the GlcNAc moiety, followed by conjugation with an alkyne-containing biotin compound. The experiment was performed based on the Click-iT O-GlcNAc Enzymatic Labeling System protocol, and the Click-iT Protein Analysis Detection Kit protocol (Life Technologies). Control experiments were carried out in parallel in the absence of the labeling enzyme GalT or UDP-GalNAz. The level of O-GlcNAcylation was determined by measuring and calculating the ratio of the intensity of the band from the input (the total protein) and the elution (the O-GlcNAcylated protein).

### PGK1 purification in HEK293T cells and enzymatic assay

Flag-tagged PGK1 protein was overexpressed in HEK293T cells. Cells were firstly lysed in the lysis buffer (50 mM Tris-HCl, pH 7.4, 150 mM NaCl, 1% Triton X-100, 5 mM thimet-G and Complete protease inhibitor cocktail), and the anti-Flag-M2 magnetic beads were used to immuno-precipitate Flag-tagged proteins. The beads were then washed twice with NETFS buffer (100 mM NaCl, 50 mM Tris-HCl pH 7.4, 5 mM EDTA, 5 mM thiamet-G and Complete protease inhibitor cocktail) containing 1% Triton X-100, and twice with 10 mL of NETFS. The Flag-tagged PGK1 was eluted with the 3× Flag peptide in NETFS buffer. The eluent was further purified and concentrated using an Amicon Ultra Centrifugal Filter (10 kDa molecular weight cutoff; Millipore) in a buffer containing 50 mM Tris-HCl pH 7.5, 100 mM KCl, 5 mM MgCl_2_ and 5% glycerol.

PGK1 activity was measured using purified Flag-tagged PGK1 in the reaction buffer containing 55 mM Tris-HCl pH 7.6, 8 mM MgCl_2_, 4 mM ATP, 0.2 mM NADH, 12 mM 3-phosphoglycerate, and 8 U of GAPDH in a total volume of 0.2 mL at room temperature. The change in absorbance at 340 nm owing to decrease of NADH was measured using BioTek Synergy Neo Multi-Mode Plate Reader (BioTek).

### PGK1 expression and purification in E. coli

PGK1 gene was cloned into pET-28b (+) expression vector and transformed into E. coli strain BL21 (DE3). The transformant were grown in LB containing kanamycin (50 μg/ml) at 37 °C until the OD_600_ of the culture was up to 0.6–0.8. Protein expression was induced at 25 °C by addition of 1 mM of IPTG for 4 h. Afterward, cells were harvested. The harvested cells were re-suspended in lysis buffer (50 mM Tris, 150 mM NaCl, pH 8.0) and disrupted by sonication. The recombinant protein was then purified by His-tag Purification Resin (Beyotime). After equilibration with the binding buffer (50 mM Tris, 150 mM NaCl, pH 8.0), lysates were loaded on the column and washed three times with the washing buffer (50 mM Tris, 150 mM NaCl, 2 mM Imidazole, pH 8.0). The bound protein was eluted with the elution buffer (50 mM Tris, 150 mM NaCl, 50 mM imidazole), and concentrated by ultrafiltration.

### Extracellular acidification rate (ECAR) and oxygen consumption rate (OCR) assay

Extracellular acidification rate (ECAR) and oxygen consumption rate (OCR) were determined using the XF96 Extracellular Flux Analyzer (Seahorse Bioscience). Cells were plated at a density of 1.0 × 10^4^ /well in an XF96 plate and incubated overnight. The media were exchanged to XF media 1 h before the assay. XF Glycolysis Stress Test Kit was used to measure the glycolytic capacity. Glucose, oligomycin and 2-deoxy glucose (2-DG) were diluted into XF media and loaded into the cartridge to achieve final concentrations of 10, 1, and 50 mM, respectively. ECAR was determined according to the manufacturer’s instructions. XF Cell Mito Stress Test Kit was used to measure cellular mitochondrial function. Oligomycin, FCCP, antimycin and rotenone were diluted into XF media and loaded into the cartridge to achieve final concentrations of 1, 1, 5, and 1 μM, respectively. OCR was determined according to the manufacturer’s instructions.

### Cell proliferation assay

A total of 1 × 10^5^ cells was seeded in 6-cm dishes. Cells in each dish were trypsinized and counted every day after seeding. Data represent the means SEM of three independent experiments.

### Cell cycle analysis

Cells were suspended in 70% ethanol and fixed overnight at 4 °C. Cells were then treated with 20 μg/mL RNase A, followed by 25 μg/mL propidium iodide (PI). The proportion of cells in G0/G1, S and G2/M phases was determined by examining the intensity of PI fluorescence with a flow cytometer (Becton Dickinson).

### Generation of stable cell lines

To deplete endogenous PGK1, the shRNA targeting sequence 5′CCGGGCAAGGATGTTCTGTTCTTGACTCGAGTCAAGAACAGAACA-TCCTTGCTTTTTTG-3′ or the corresponding scramble sequence 5′-CCGGGACTACGTTCGAATTGTCGTTCTGCAGAACGACAATTCGAACGTAG-TCTTTTTTG-3′ was inserted into pLenti-FlagN-shRNA vector, which enables the knockdown of the endogenous gene and the simultaneous expression of an exogenous gene. The rescued PGK1 sequence (flag-tagged WT or T255V PGK1) was generated resistant to the PGK1 shRNA via silent mutations (lower case: 5′-GtAAGGATGTcCTGTTCTTGA-3′). Cells were infected with the lentiviruses produced from these constructs and selected for two weeks with puromycin.

### Site mapping of PGK1 O-GlcNAcylation

Flag-tagged PGK1 and HA-tagged OGT were co-transfected in HEK293T cells using Lipofectamine 2000 according to the manufacturer’s protocol. After 72 h, Flag-tagged PGK1 was purified from cells using anti-Flag-M2 magnetic beads. The bound protein was eluted in a buffer (4% SDS and 100 mM Tris-HCl, pH 8.0), and subjected to SDS-PAGE and stained with Bio-Safe Coomassie blue R250 Stain. The PGK1 protein band was then excised from the gel and manually digested in-gel with trypsin overnight. The extracted peptides were further purified by reverse-phase HPLC (Agilent 1100) using a preparative reverse-phase column (Agilent Eclipse XDB-C18; 5μm, 9.4 × 250 mm) and a gradient of 5–30% B buffer over 20 min at 4 ml min^−1^ (A buffer, 0.5% aqueous AcOH; B buffer, 100% MeCN). Fractions eluted between 5–12 min were collected, lyophilized and subjected to nanoLC-LTQ-CID/ETD-MS analysis on an LTQ-Orbitrap Velos. Data search were performed by Proteome Discovery (MASCOT search engine, version 1.3) with O-GlcNAc (Ser/Thr) set as a variable modification.

### Cell stress analysis

At 48 h after transfection with Flag-tagged PGK1, cells were treated with 0.5 mM H_2_O_2_, 1 mM H_2_O_2_, 0.5 mM diamide (a thiol-oxidizing compound, Sigma-Aldrich) or 1 mM diamide for 30 min, respectively.

Hypoxic treatment was performed in a sealed hypoxia chamber (Proox Model 110, BioSpherix) filled with 1% O_2_, 5% CO_2_, and 94% N_2_ at 37 °C and 70% cell confluency for 12 h. For glucose treatment assays, HEK293T cells were cultured in low glucose DMEM medium (5 mM, Hyclone) or high glucose DMEM medium (25 mM) for 24 h after transfected with the Flag-PGK1 construct.

### LC–MS for metabolite analysis

A total of 1 × 10^7^ cells was vortex mixed in 1 mL precooled solution consisting of methanol/acetonitrile/water (2:2:1, v/v/v). After sonication, proteins were precipitated at −20 °C for 1 h. Supernatants separated by centrifugation were proceeded to vacuum drying and then re-dissolved in 100 μL acetonitrile/water solution (1:1, v/v). After centrifugation, the supernatant was diluted five times for detection.

Chromatographic separation was performed on an Agilent 1290 Infinity LC (Agilent). Samples were stocked in an autosampler at 4 °C, and the mobile phase composition was a mixture of 15 mM ammonium acetate (A) and acetonitrile (B) using a gradient program from 40% to 90% for 5 min. The flow rate was 300 μL/min and the injection volume was 4 μL. The profiling of representative metabolites in glycolysis, TCA, PPP, and SSP were carried out on a 5500 QTRAP tandem mass spectrometer (AB SCIEX) equipped with an electrospray source operating in the negative-ion multiple-reaction monitoring mode. The ion source settings were as follows: source temperature, 450 °C; ion source gas1, 45; ion source gas2, 45; curtain gas, 30; ionsapary voltage floating, −4500 V.

Data were acquired and processed using Multiquant software. Peak areas of individual metabolites were normalized against the total cell number.

### Measurement of glucose uptake, lactate production, ATP, and ADP levels

Glucose uptake was measured using a glucose uptake colorimetric assay kit (BioVision) according to the manufacturer’s protocol. Briefly, cells were seeded at a density of 2,000 cells per well in a 96-well plate. Cells were starved in serum free culture medium overnight. Cells were then washed with phosphate-buffered saline (PBS) and incubated with 100 μL Krebs-Ringer-Phosphate-HEPES (KRPH) buffer containing 2% BSA for 40 min. Ten microliters of 10 mM 2-deoxyglucose was then added and incubated for 20 min. Cells were proceeded to oxidation step to generate NADPH, which is determined by an enzymatic recycling amplification reaction. The absorbance at 412 nm was recorded using a BioTek microplate reader and normalized to protein concentration.

The culture medium was collected for the measurement of lactate release from cells. Lactate levels were determined using a lactate colorimetric assay kit (BioVision) according to the manufacturer’s protocol. The absorbance at 450 nm was recorded using a BioTek microplate reader and normalized to protein concentration.

ATP and ADP levels were measured using an ATP/ADP colorimetric assay kit (BioVision) according to the manufacturer’s protocol. The absorbance at 450 nm was recorded using a BioTek microplate reader and normalized to protein concentration.

### Determination of PGK1 half-life

GK1 WT or T255V rescue HT-29 cells were treated with 50 μM cycloheximide (CHX) to inhibit new protein synthesis. After treatment, cells were harvested at the indicated time points (0, 10, 20, 40 or 80 min). PGK1 levels were then detected by western blotting with anti-PGK1 antibody, and the relative half-life was calculated.

### Determination of cellular levels of reactive oxygen species

Intercellular reactive oxygen species (ROS) were determined by staining with DCFH-DA. Cells were incubated with 10 μM DCFH-DA at 37 °C for 30 min. After incubation with the fluorochrome, cells were washed with PBS and immediately detected by flow cytometry.

### Xenograft model in nude mice

The procedure of establishing HT-29 xenografts in nude mice was performed under the animal experiment protocol of the Zhejiang University Laboratory Animal Centre (Hangzhou, China) and approved by the institution. Briefly, 4 × 10^6^ HT-29 cells (stably expressing scramble shRNA, shPGK1, rescued with PGK1 WT, with PGK1 T255V) cells were subcutaneously injected into the flanks of 6-week-old male nude BALB/c mice. Tumour growth was monitored every 3 days over a 7-week period. At the end of the seventh week, the tumours were harvested and weighed.

### Statistical analysis

Data are presented as the means ± standard deviations (SD). Statistical analyses were performed by one-way analysis of variance (ANOVA) followed by Tukey’s post hoc test. A value of *P* < 0.05 was considered statistically significant.

### Reporting summary

Further information on research design is available in the Nature Research Reporting Summary linked to this article.

## Supplementary information

Supplementary Information

Reporting Summary

## Data Availability

The data that support the findings of this study are available from the corresponding author upon request. The source data underlying Figs. 1a, b, c, d, e, f, g; 2a, b, c, d, e, g; 3a, b, c; 4a, b, c, d; 5b, c, d; 6a, b, d, e, f, g; 7a, b, c, d, e, f; 8a, b, c, d and e, and Tables 1 and 2, and Supplementary Figs. 1, 2, 3, 4, 6a, b; 7, 8a, b; 9a, b; 10b, c, d, and 11, and Supplementary Tables 1–4 are provided as a Source Data file. The mass spectrometry proteomics data are available via ProteomeXchange with identifier PXD015659.

## Source data

Source Data

## References

[1] Hirschey MD (2015). Dysregulated metabolism contributes to oncogenesis. Semin Cancer Biol..

[2] Intlekofer AM, Finley LWS (2019). Metabolic signatures of cancer cells and stem cells. Nat. Metab..

[3] Schworer S, Vardhana SA, Thompson CB (2019). Cancer metabolism drives a stromal regenerative response. Cell Metab..

[4] Tekade RK, Sun X (2017). The Warburg effect and glucose-derived cancer theranostics. Drug Disco. Today.

[5] Liberti MV, Locasale JW (2016). The Warburg effect: how does it benefit cancer cells?. Trends Biochem Sci..

[6] Yang X, Qian K (2017). Protein O-GlcNAcylation: emerging mechanisms and functions. Nat. Rev. Mol. Cell Biol..

[7] Joiner CM, Li H, Jiang J, Walker S (2019). Structural characterization of the O-GlcNAc cycling enzymes: insights into substrate recognition and catalytic mechanisms. Curr. Opin. Struct. Biol..

[8] Banerjee PS, Lagerlof O, Hart GW (2016). Roles of O-GlcNAc in chronic diseases of aging. Mol. Asp. Med..

[9] Leney AC, El Atmioui D, Wu W, Ovaa H, Heck AJR (2017). Elucidating crosstalk mechanisms between phosphorylation and O-GlcNAcylation. Proc. Natl. Acad. Sci. USA.

[10] Zeidan Q, Hart GW (2010). The intersections between O-GlcNAcylation and phosphorylation: implications for multiple signaling pathways. J. Cell Sci..

[11] Wani WY, Chatham JC, Darley-Usmar V, McMahon LL, Zhang J (2017). O-GlcNAcylation and neurodegeneration. Brain Res. Bull..

[12] Slawson C, Copeland RJ, Hart GW (2010). O-GlcNAc signaling: a metabolic link between diabetes and cancer?. Trends Biochem. Sci..

[13] Dassanayaka S, Jones SP (2014). O-GlcNAc and the cardiovascular system. Pharm. Ther..

[14] Ferrer CM, Sodi VL, Reginato MJ (2016). O-GlcNAcylation in Cancer Biology: Linking Metabolism and Signaling. J. Mol. Biol..

[15] Ma Z, Vosseller K (2014). Cancer metabolism and elevated O-GlcNAc in oncogenic signaling. J. Biol. Chem..

[16] Hu H (2017). Acetylation of PGK1 promotes liver cancer cell proliferation and tumorigenesis. Hepatology.

[17] Li X (2016). Mitochondria-translocated PGK1 functions as a protein kinase to coordinate glycolysis and the TCA Cycle in tumorigenesis. Mol. Cell.

[18] Zhang Y (2018). Macrophage-associated PGK1 phosphorylation promotes aerobic glycolysis and tumorigenesis. Mol. Cell.

[19] Wang S (2015). Insulin and mTOR pathway regulate HDAC3-mediated deacetylation and activation of PGK1. PLoS Biol..

[20] DeBerardinis R. J., Mancuso A., Daikhin E., Nissim I., Yudkoff M., Wehrli S., Thompson C. B. (2007). Beyond aerobic glycolysis: Transformed cells can engage in glutamine metabolism that exceeds the requirement for protein and nucleotide synthesis. Proceedings of the National Academy of Sciences.

[21] Le A (2012). Glucose-independent glutamine metabolism via TCA cycling for proliferation and survival in B cells. Cell Metab..

[22] Fan J (2013). Glutamine-driven oxidative phosphorylation is a major ATP source in transformed mammalian cells in both normoxia and hypoxia. Mol. Syst. Biol..

[23] Hitosugi T (2013). Tyr26 phosphorylation of PGAM1 provides a metabolic advantage to tumours by stabilizing the active conformation. Nat. Commun..

[24] Lv L (2013). Mitogenic and oncogenic stimulation of K433 acetylation promotes PKM2 protein kinase activity and nuclear localization. Mol. Cell.

[25] Yi W (2012). Phosphofructokinase 1 glycosylation regulates cell growth and metabolism. Science.

[26] Groves JA, Lee A, Yildirir G, Zachara NE (2013). Dynamic O-GlcNAcylation and its roles in the cellular stress response and homeostasis. Cell Stress Chaperones.

[27] Guzy RD (2005). Mitochondrial complex III is required for hypoxia-induced ROS production and cellular oxygen sensing. Cell Metab..

[28] Anastasiou D (2011). Inhibition of pyruvate kinase M2 by reactive oxygen species contributes to cellular antioxidant responses. Science.

[29] Rao X (2015). O-GlcNAcylation of G6PD promotes the pentose phosphate pathway and tumor growth. Nat. Commun..

[30] Qian Y, Wang X, Li Y, Cao Y, Chen X (2016). Extracellular ATP a new player in cancer metabolism: NSCLC cells internalize ATP in vitro and in vivo using multiple endocytic mechanisms. Mol. Cancer Res..

[31] Finder D. R. & Hardin C. D. Transport and metabolism of exogenous fumarate and 3-phosphoglycerate in vascular smooth muscle. *Mol. Cell Biochem*. **195**, 113–121 (1999). 10.1023/a:1006976432578 10395075

[32] van der Laarse SAM, Leney AC, Heck AJR (2018). Crosstalk between phosphorylation and O-GlcNAcylation: friend or foe. FEBS J..

[33] Yi M (2019). 6-Phosphofructo-2-kinase/fructose-2,6-biphosphatase 3 and 4: A pair of valves for fine-tuning of glucose metabolism in human cancer. Mol. Metab..

[34] Vander Heiden MG, DeBerardinis RJ (2017). Understanding the intersections between metabolism and cancer biology. Cell.

[35] Sun L, Suo C, Li ST, Zhang H, Gao P (2018). Metabolic reprogramming for cancer cells and their microenvironment: beyond the Warburg Effect. Biochim Biophys. Acta Rev. Cancer.

[36] Pavlova NN, Thompson CB (2016). The emerging hallmarks of cancer metabolism. Cell Metab..

[37] Wang Y (2017). O-GlcNAcylation destabilizes the active tetrameric PKM2 to promote the Warburg effect. Proc. Natl. Acad. Sci. USA.

[38] Li X (2018). Nuclear PGK1 alleviates ADP-dependent inhibition of CDC7 to promote DNA replication. Mol. Cell.

[39] Luengo A, Gui DY, Vander Heiden MG (2017). Targeting metabolism for cancer therapy. Cell Chem. Biol..

[40] Anderson NM, Mucka P, Kern JG, Feng H (2018). The emerging role and targetability of the TCA cycle in cancer metabolism. Protein Cell.

[41] Sullivan LB, Gui DY, Vander Heiden MG (2016). Altered metabolite levels in cancer: implications for tumour biology and cancer therapy. Nat. Rev. Cancer.

