# Supplementary material for: O-GlcNAcylation of PGK1 coordinates glycolysis and TCA cycle to promote tumor growth
Source: Nat Commun. 2020 Jan 7;11:36. doi: 10.1038/s41467-019-13601-8 (PMC6946671; doi:10.1038/s41467-019-13601-8)
Supplement: Supplementary file 1 — Supplementary Information [file 41467_2019_13601_MOESM1_ESM.doc]

**Supplementary Information**

**O-GlcNAcylation of PGK1 Coordinates Glycolysis and TCA Cycle to Promote Tumor Growth**

**Nie *et al.*2019**

**
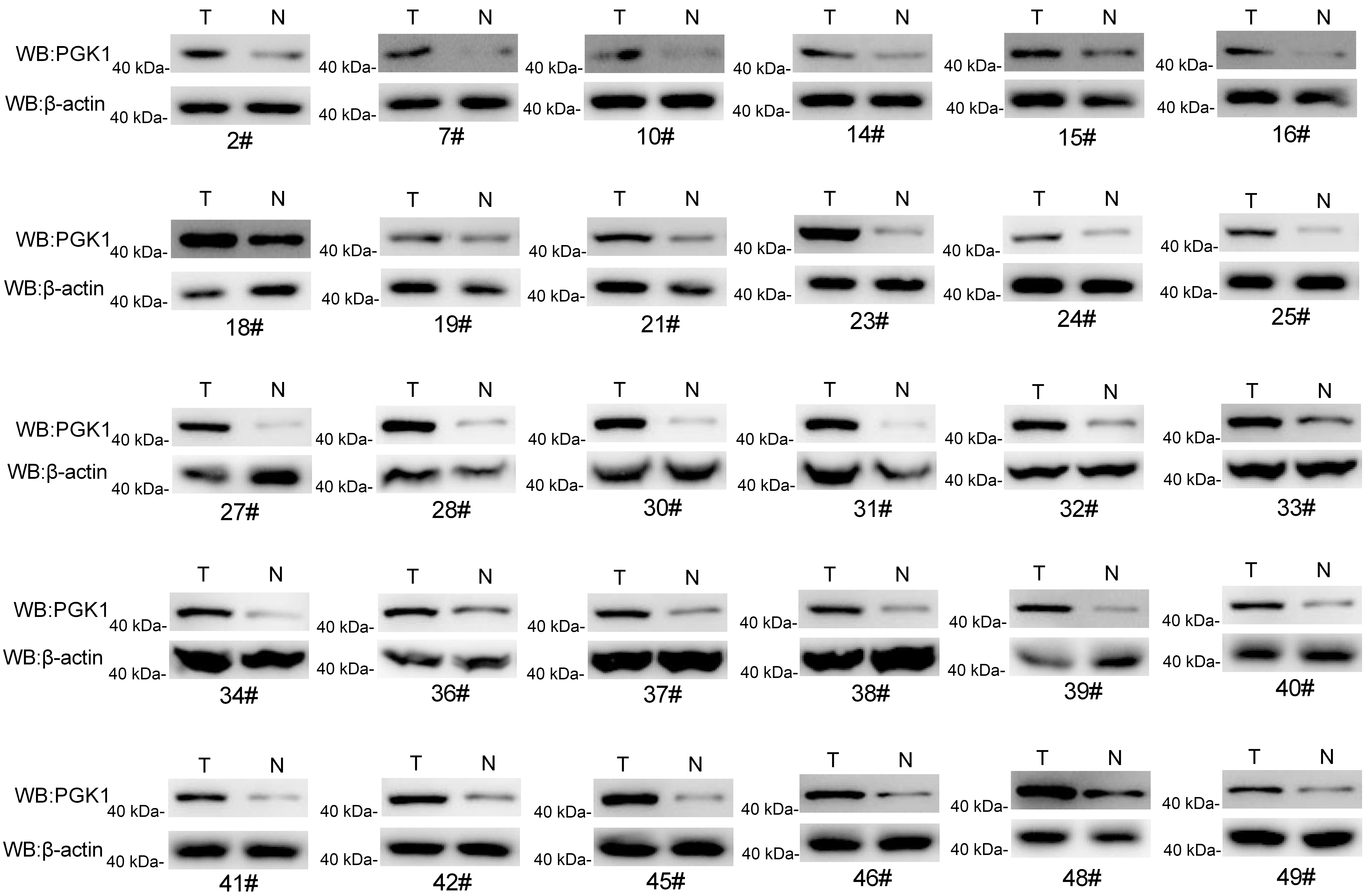
**

**Supplementary Fig 1.** Immunoblotting analysis of PGK1 protein expression in the remaining 30 paired human colon tumor (T) tissues and the matching adjacent normal (N) tissues. Source data are provided as a Source Date file.

**
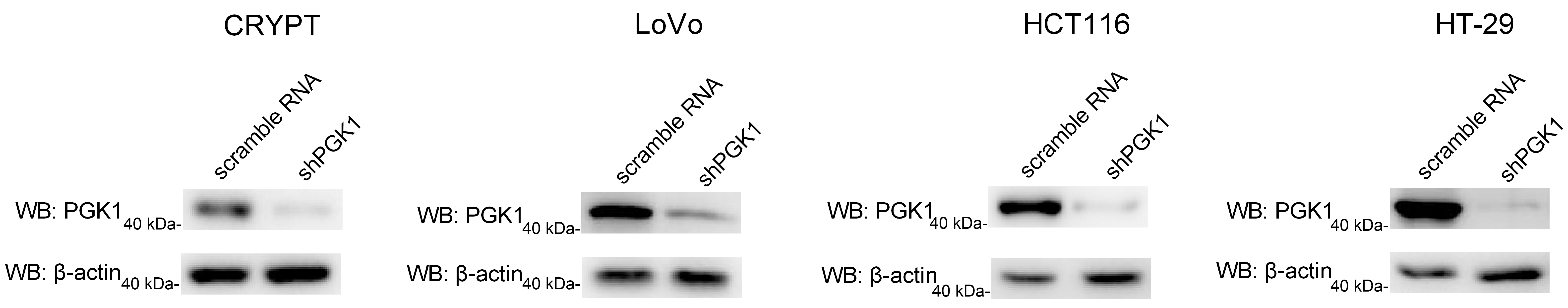
**

**Supplementary Fig 2.** Immunoblotting analysis of PGK1 depletion with small hairpin RNA (shRNA) in CRYPT, LoVo, HCT-116 and HT-29 cells. Source data are provided as a Source Date file.

**
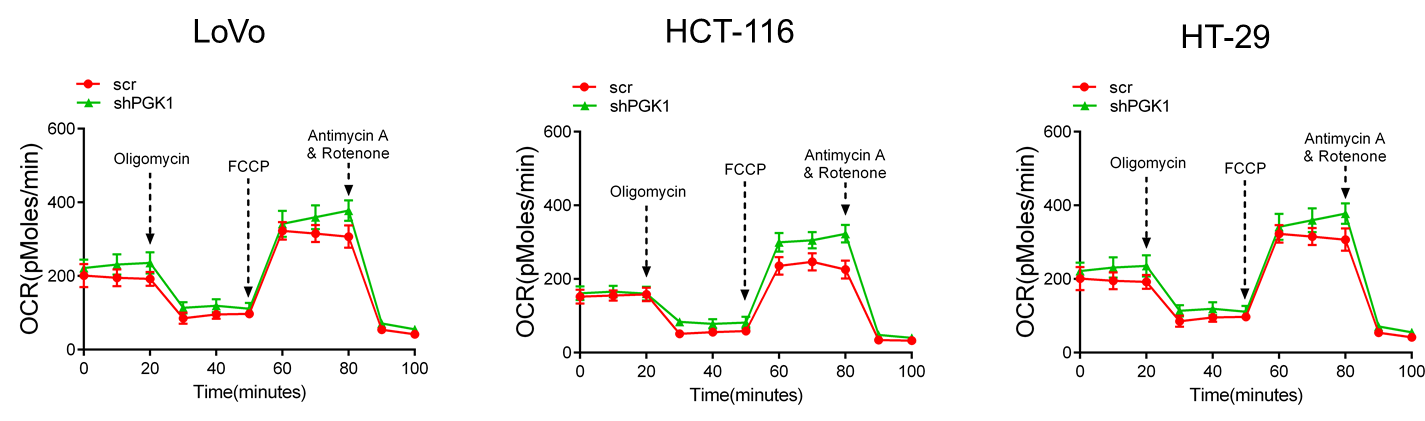
**

**Supplementary Fig 3.** The oxygen consumption rate (OCR) in LoVo, HCT-116 and HT-29 cells infected with control shRNA or shPGK1. Error bars denote the means ± standard deviations (SD). Source data are provided as a Source Date file.

**
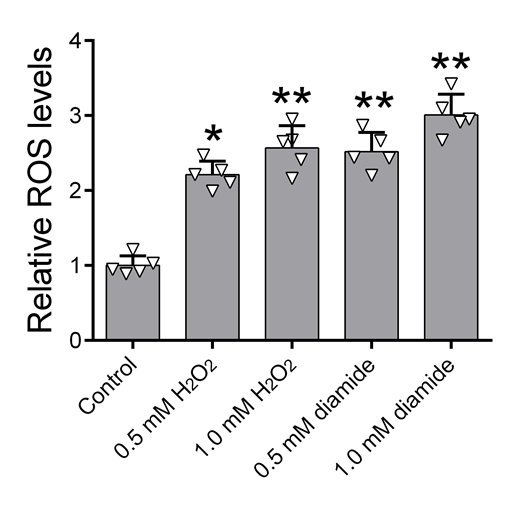
**

**Supplementary Fig 4.** Increase of reactive oxygen species (ROS) levels in HT-29 cells by treatment with H_2_O_2_ or diamide. Error bars denote the means ± standard deviations (SD). Statistical analyses were performed by one-way analysis of variance (ANOVA) followed by Tukey's post hoc test (*P< 0.05, **P<0.01). Source data are provided as a Source Date file.

**
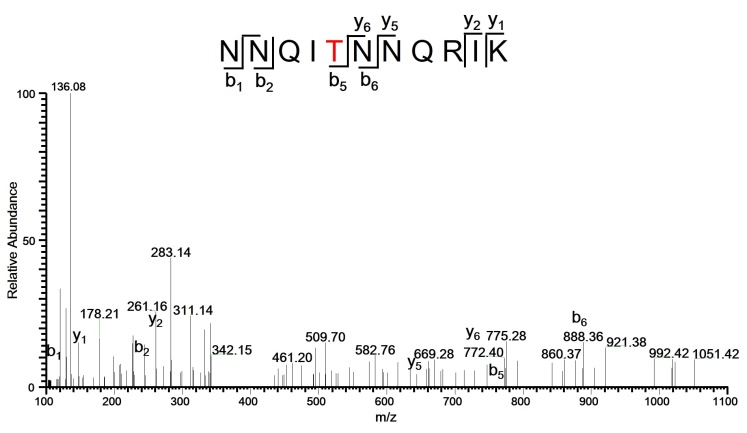

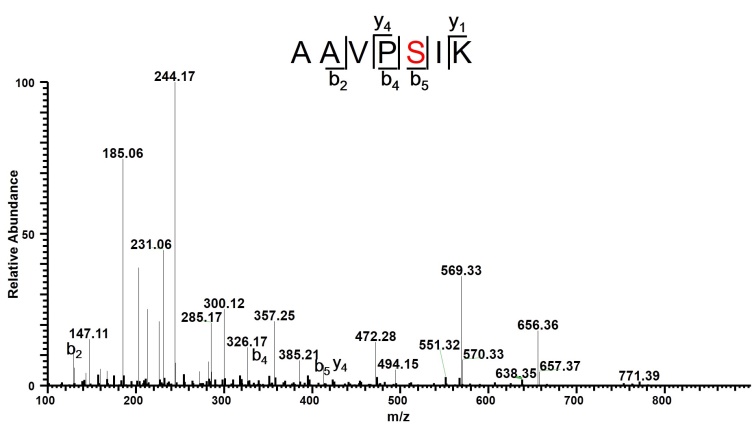

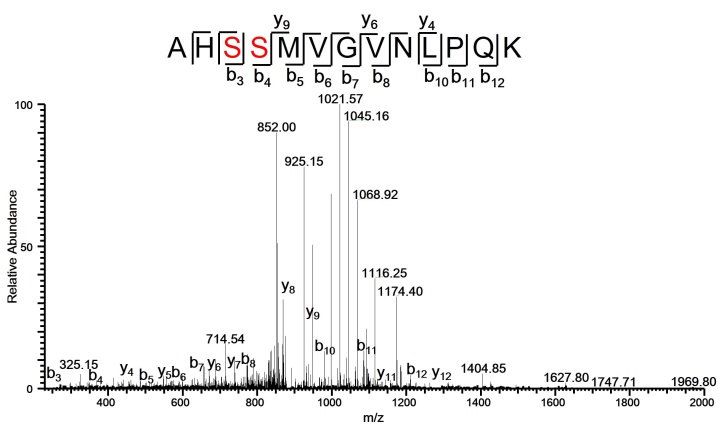

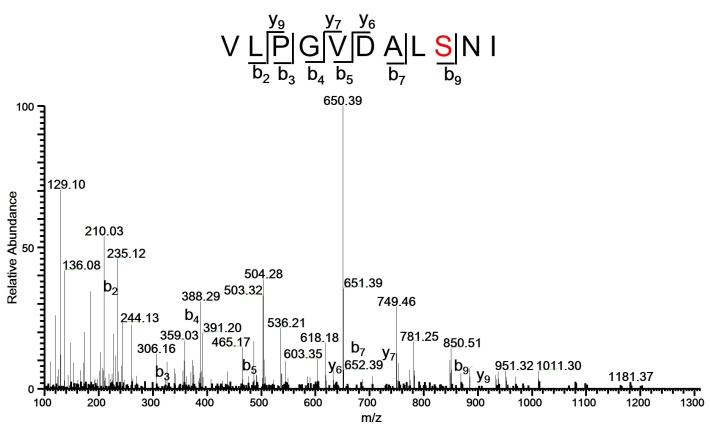
**

T35

S46

S174/S175

S415

**Supplementary Fig 5.** Mapping the O-GlcNAcylation sites on PGK1 using nanoLC-LTQ-CID/MS.


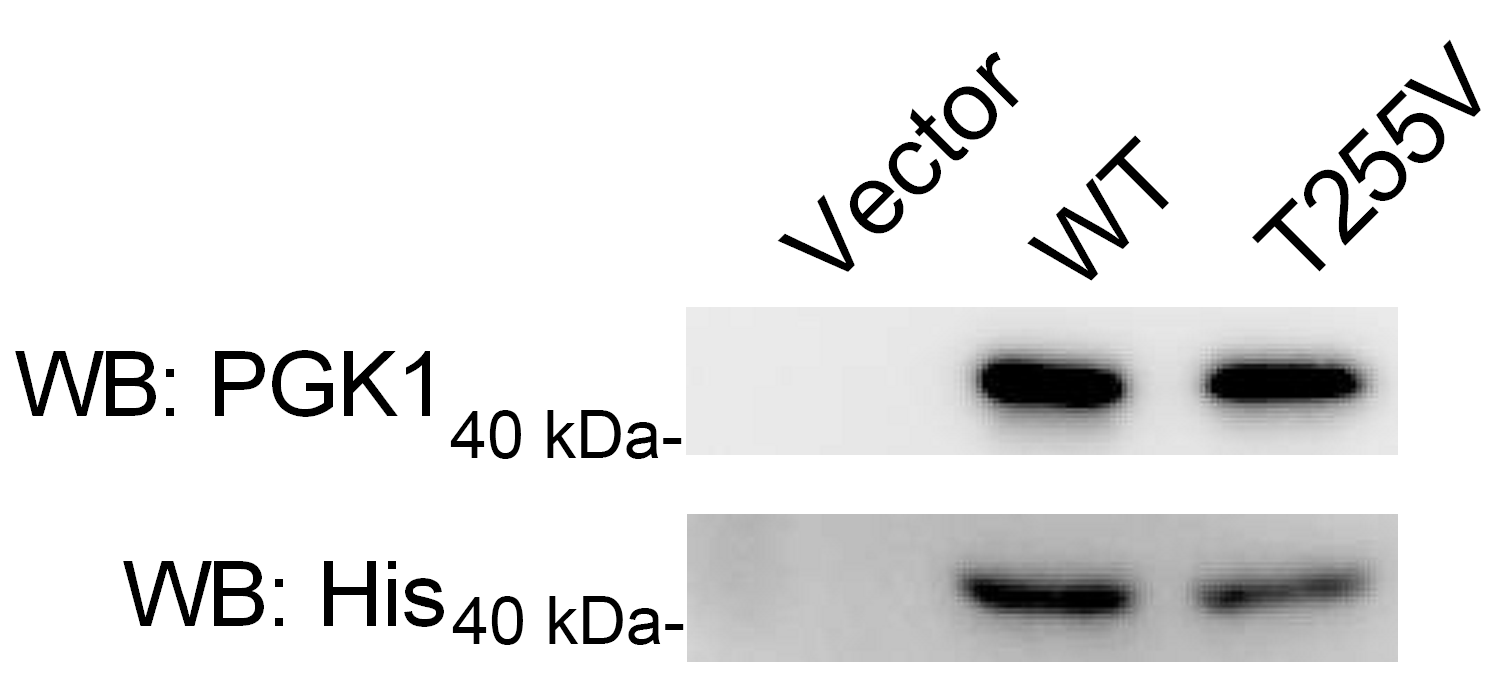

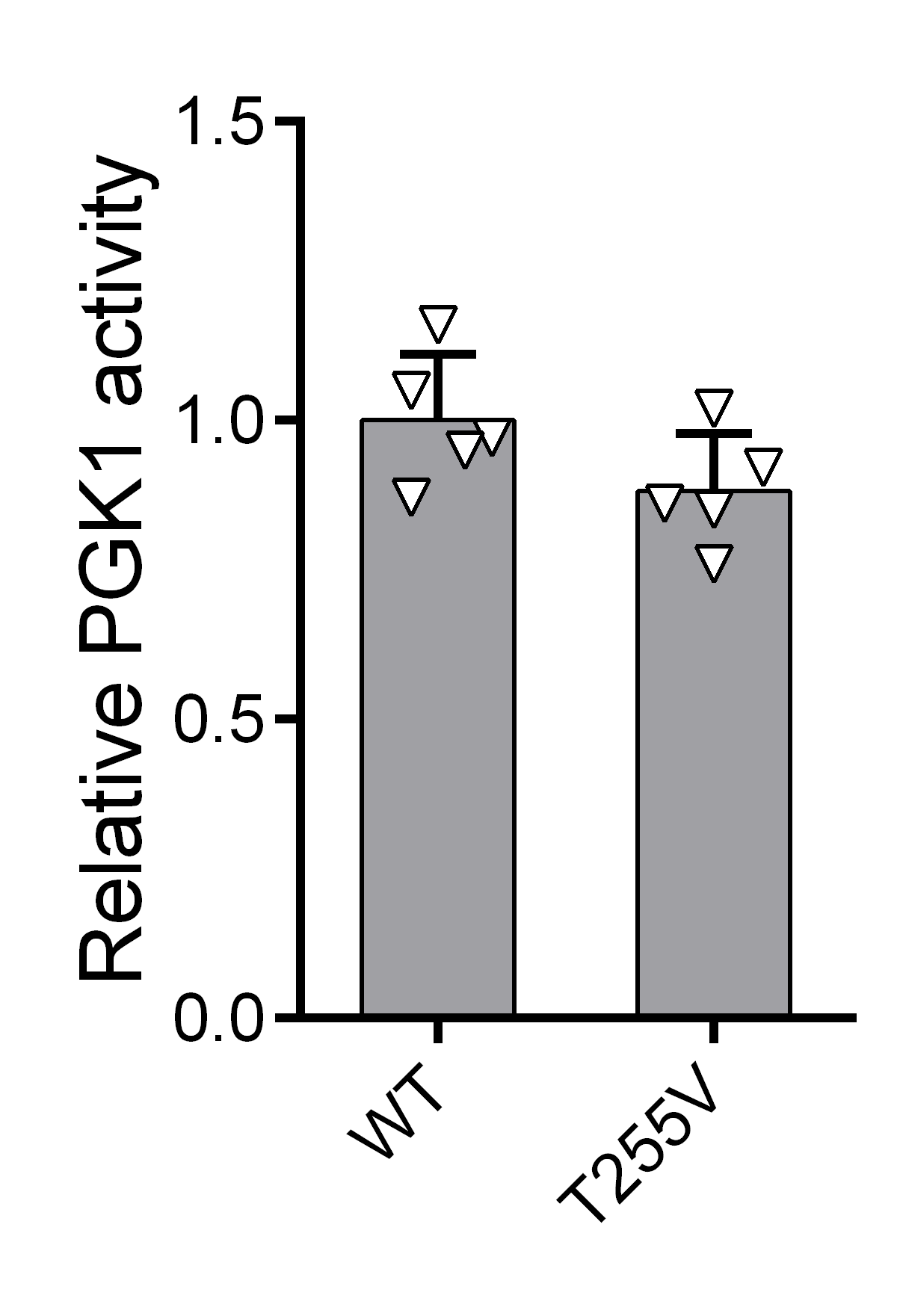


**Supplementary Fig 6.** Expression of PGK1 WT or T255V in E. coli. Error bars denote the means ± standard deviations (SD). Source data are provided as a Source Date file.

**
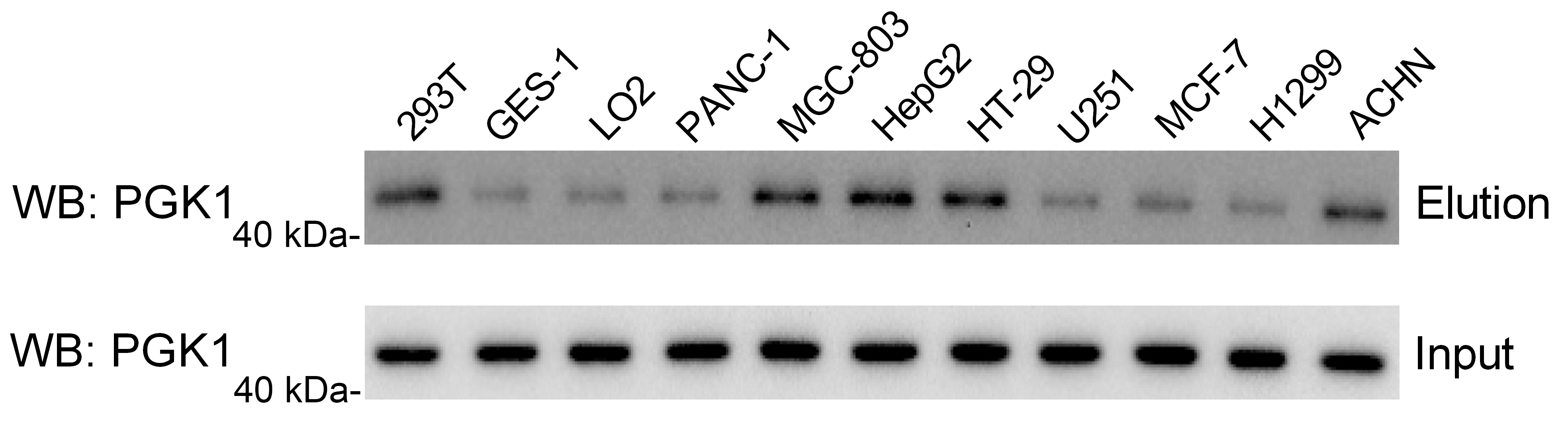
**

**Supplementary Fig 7.** Immunoblotting analysis of levels of PGK1 glycosylation in different cell lines. Source data are provided as a Source Date file.

**
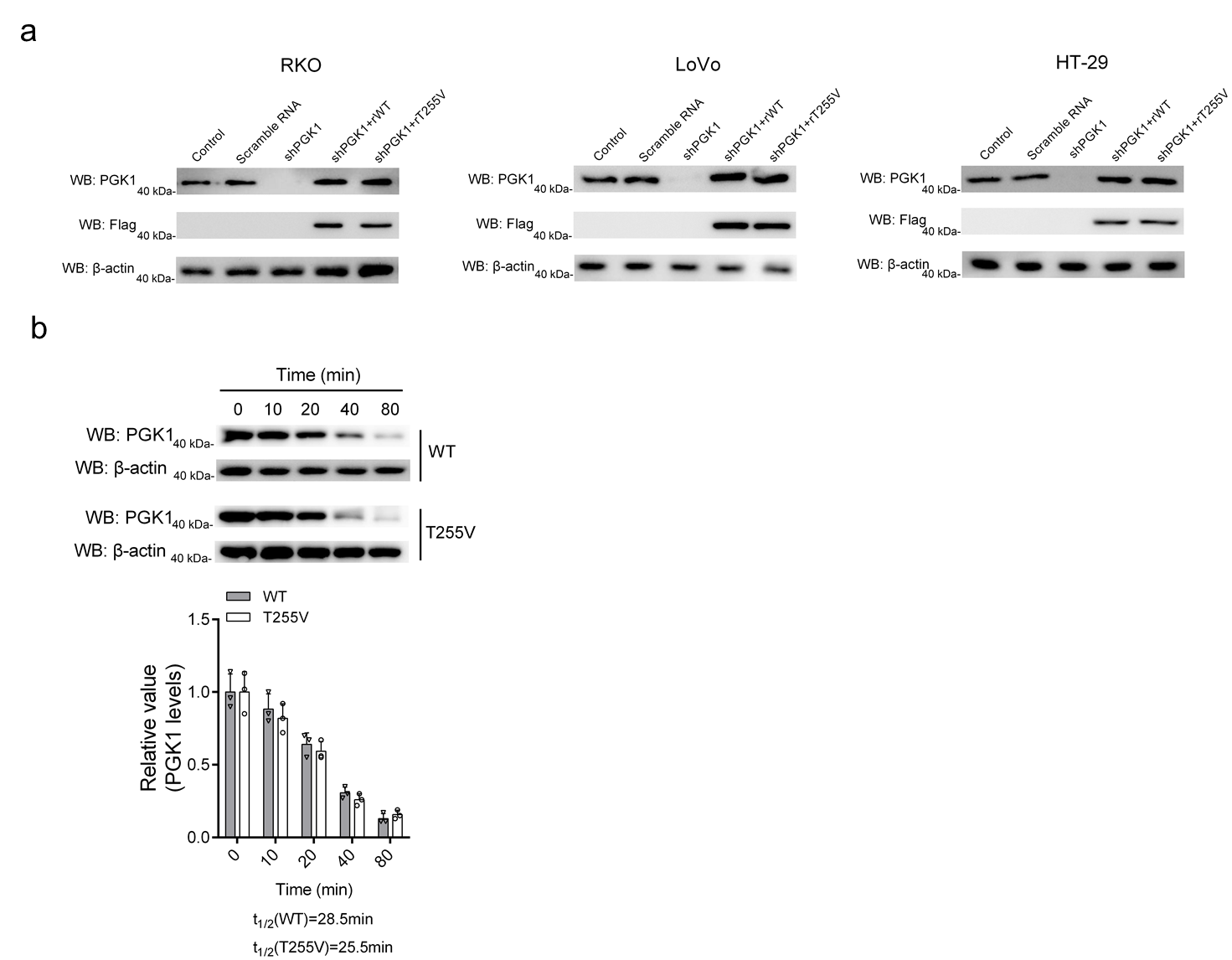
**

**Supplementary Fig 8. a,** Generation of WT PGK1 and T255V PGK1 rescue cell lines. Endogenous PGK1 was depleted with PGK1 shRNA. shRNA-resistant Flag-tagged WT or T255V PGK1 was stably expressed in RKO, LoVo, and HT-29 cells. **b**, Half-life of PGK1 WT and T255V in cells. Error bars denote the means ± standard deviations (SD). Source data are provided as a Source Date file.

**
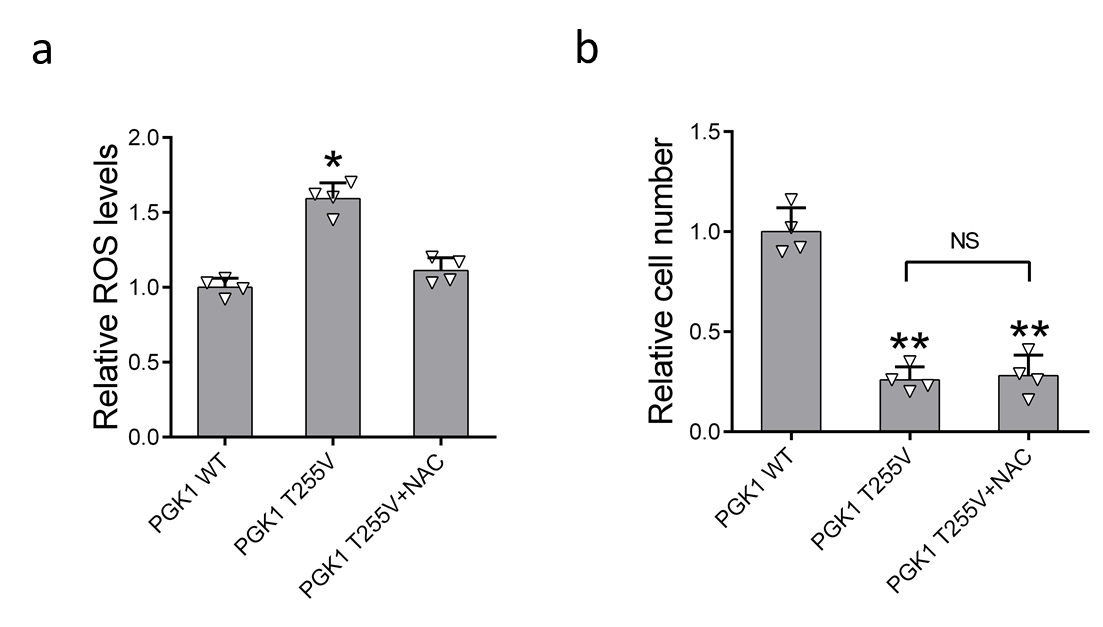
**

**Supplementary Fig 9. a,** Treatment with N-acetyl cysteine (NAC) reduced ROS levels in T255V rescue HT-29 cells. **b,** Treatment with N-acetyl cysteine (NAC) failed to rescue the proliferation in T255V rescue HT-29 cells. Error bars denote the means ± standard deviations (SD). Statistical analyses were performed by one-way analysis of variance (ANOVA) followed by Tukey's post hoc test (*P< 0.05, **P<0.01, NS=no significant difference). Source data are provided as a Source Date file.

**
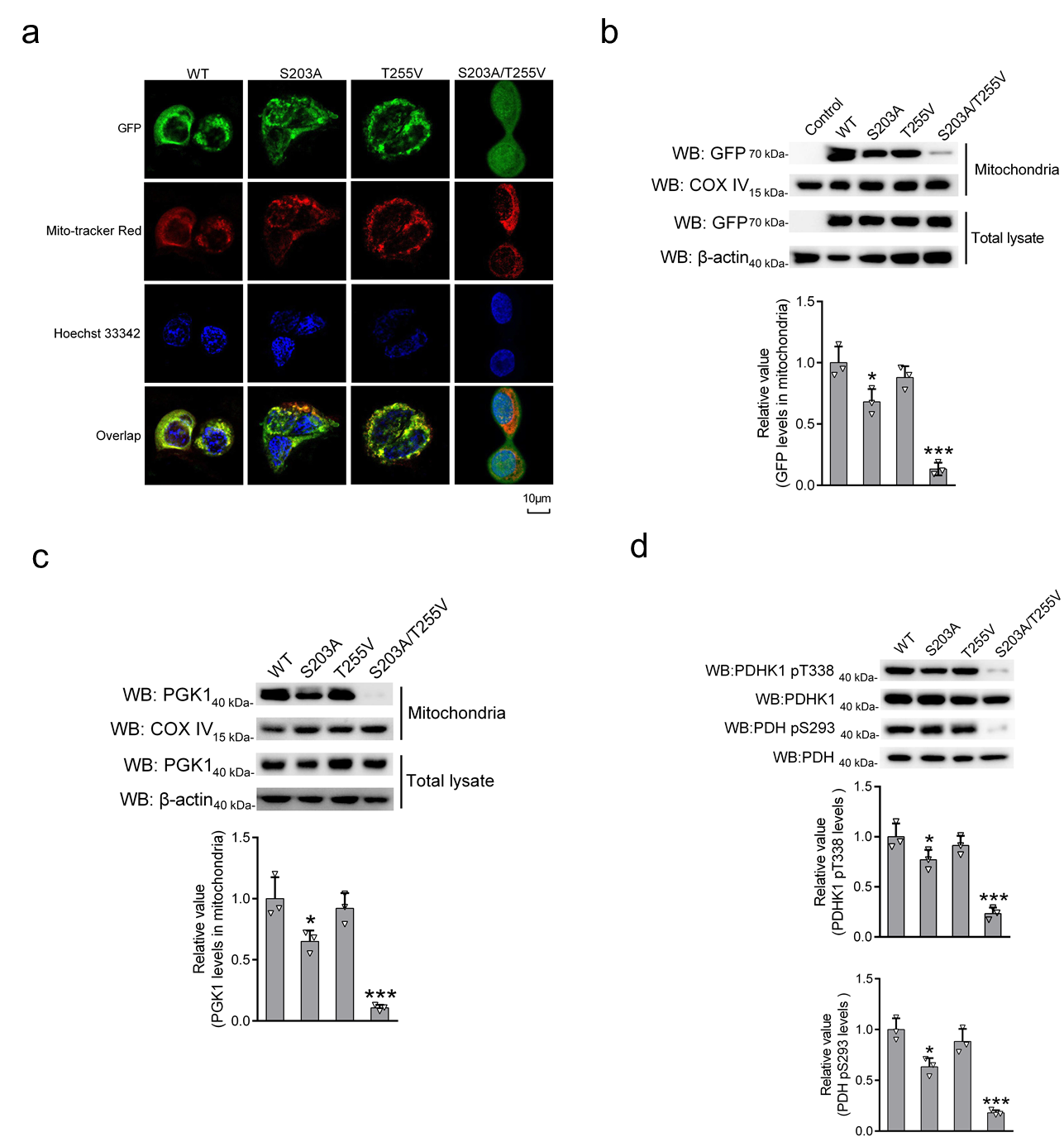
**

**Supplementary Fig 10. a,** Cellular distribution of PGK1 as determined by immunostaining in the presence of hypoxia. HT-29 cells were transfected with GFP-fused PGK1 WT or T255V construct. Cells were co-stained with MitoTracker red, a fluorescent marker for mitochondria. **b** and **c**, Immunoblotting analysis of cellular distribution of PGK1 WT or T255V. **d,** Immunoblotting analysis of PDHK1 and PDH phosphorylation in mitochondria. Mitochondrial fractions were prepared and immunoblotted with indicated antibodies. Total cell lysates were used as a control. Each data point was the average of at least three independent measurements. Error bars denote the means ± standard deviations (SD). Statistical analyses were performed by one-way analysis of variance (ANOVA) followed by Tukey's post hoc test (*P< 0.05, **P<0.01, ***P<0.001). Source data are provided as a Source Date file.

**
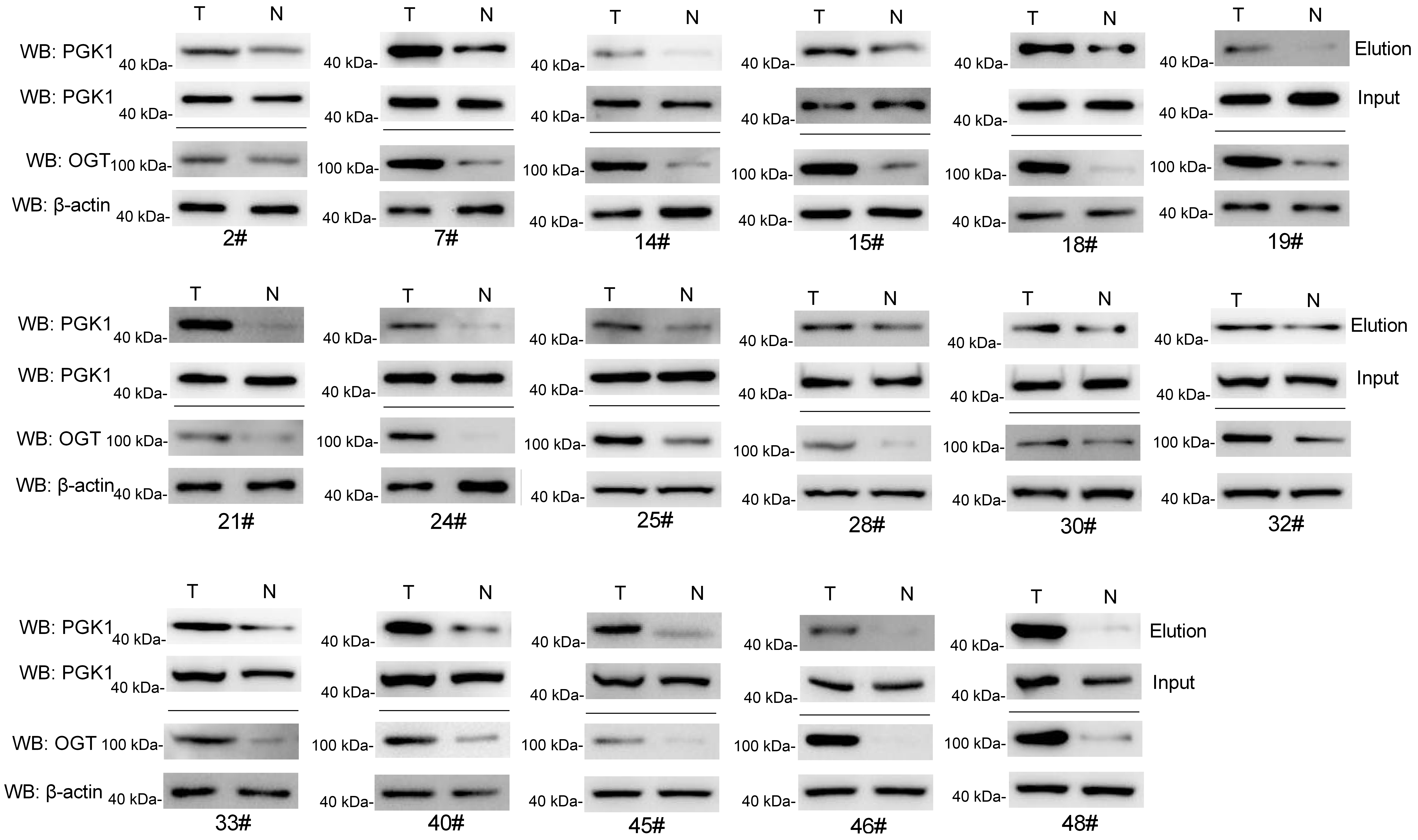
**

**Supplementary Fig 11.** Immunoblotting analysis of PGK1 glycosylation and OGT expression in other 17 pairs human colon tumor (T) tissues and the matching adjacent normal (N) tissues. Source data are provided as a Source Date file.

**Supplementary Table 1. Steady-state kinetics of 3-PG**

| PGK1 | K_cat_(s^-1^) | K_m 3-PG_(μM) | K_cat_/ K_m 3-PG_ |
| --- | --- | --- | --- |
| WT | 23.46±1.80 | 4.77±0.35 | 4.94±0.61 |
| WT+OGT | 52.09±2.70 | 4.15±0.15 | 12.55±0.21 |
| T255V | 16.84±1.99 | 7.50±1.13 | 2.30±0.58 |
| T255V+OGT | 16.43±2.93 | 7.73±1.06 | 2.12±0.23 |

Data are represented as mean ± SD. Source data are provided as a Source Date file.

**Supplementary Table 2. Steady-state kinetics of ADP**

| PGK1 | K_cat_(s^-1^) | K_m ADP_(μM) | K_cat_/ K_m ADP_ |
| --- | --- | --- | --- |
| WT | 152.74±8.31 | 7.69±1.36 | 20.28±3.52 |
| WT+OGT | 316.95±19.51 | 3.59±0.72 | 90.14±13.90 |
| T255V | 116.49±10.56 | 13.92±2.60 | 8.60±1.92 |
| T255V+OGT | 120.16±5.02 | 12.37±1.73 | 9.81±1.17 |

Data are represented as mean ± SD. Source data are provided as a Source Date file.

**Supplementary Table 3. Steady-state kinetics of 3-PG (PGK1 was expressed in E.coli)**

| PGK1 | K_cat_(s^-1^) | K_m 3-PG_(μM) | K_cat_/ K_m 3-PG_ |
| --- | --- | --- | --- |
| WT | 11.73±1.35 | 8.61±0.79 | 1.37±0.14 |
| T255V | 10.86±0.97 | 8.95±0.26 | 1.21±0.13 |

Data are represented as mean ± SD. Source data are provided as a Source Date file.

**Supplementary Table 4. Steady-state kinetics of ADP (PGK1 was expressed in E.coli)**

| PGK1 | K_cat_ (s^-1^) | K_m ADP_ (μM) | K_cat_/ K_m ADP_ |
| --- | --- | --- | --- |
| WT | 96.32±5.52 | 16.27±0.60 | 5.93±0.48 |
| T255V | 88.97±3.83 | 17.17±0.84 | 5.20±0.46 |

Data are represented as mean ± SD. Source data are provided as a Source Date file.
